# Topological Insulator Bi_2_Te_3_ Anode for Aqueous Aluminum‐Ion Batteries: Unveiling the Role of Hydronium Ions

**DOI:** 10.1002/advs.202507255

**Published:** 2025-07-06

**Authors:** Puja De, Petr Lazar, Michal Otyepka, Martin Pumera

**Affiliations:** ^1^ Advanced Nanorobots & Multiscale Robotics Laboratory, Faculty of Electrical Engineering and Computer Science VSB – Technical University of Ostrava 17. listopadu 2172/15 Ostrava 70800 Czech Republic; ^2^ Regional Centre of Advanced Technologies and Materials The Czech Advanced Technology and Research Institute (CATRIN) Palacký University Olomouc Šlechtitelů 27 Olomouc 779 00 Czech Republic; ^3^ IT4Innnovations VSB‐Technical University of Ostrava 17. listopadu 2172/15 Ostrava‐Poruba 708 00 Czech Republic; ^4^ Future Energy and Innovation Laboratory Central European Institute of Technology Brno University of Technology Purkyňova 123 Brno 61200 Czech Republic; ^5^ Department of Medical Research China Medical University Hospital China Medical University No. 91 Hsueh‐Shih Road Taichung 40402 Taiwan

**Keywords:** anode, aqueous batteries, diffusion barrier, hydronium ion, quantum material

## Abstract

The primary scientific challenge in advancing aqueous aluminum‐ion batteries (AAIBs) is achieving reversible plating/stripping of the Al metal anode, limited by its low deposition potential (−1.667 V vs SHE) and surface passivation in the aqueous electrolyte. To address this issue, polypyrrole (PPy) decorated topological quantum insulator (Bi_2_Te_3_@PPy) is introduced as a novel anode in AAIBs. Benefiting from the interconnected PPy network and the gap‐free metallic surface state of Bi_2_Te_3_, the Bi_2_Te_3_@PPy anode enables a remarkable discharge capacity of 438 mAh g^−1^ at a current rate of 0.5 A g^−1^. It also maintains a strong discharging plateau even at a higher current rate of 10 A g^−1^, outperforming most electrode materials reported so far for AAIBs. The role of the topological surface states of Bi_2_Te_3_ in enhancing the ion migration rate is validated by comparing its performance across various morphologies. Ex situ studies and computational analysis reveal that in aqueous systems, Al^3+^ is not the sole species responsible for charge storage. Instead, hydronium ions (H_3_O^+^) significantly contribute to storing the charges through intercalation into the crystal lattice. Overall, this study pioneers a new approach for developing advanced Al metal‐free AAIBs and provides deeper insights into the charge storage mechanisms in aqueous electrolytes.

## Graphical abstract

This work introduces Bi_2_Te_3_, a novel topological quantum insulator, as an advanced anode for aqueous aluminum‐ion batteries (AAIBs). The study highlights the pivotal role of hydronium ions, generated through the hydrolysis of hydrated aluminum ions, in the charge storage process. Hence, these findings open new avenues for advancing AAIB technology and provide a deeper understanding of ion storage dynamics in aqueous media.

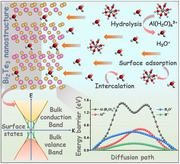

## 1 Introduction

Considering global energy storage requirements, there is an urgent need to develop a clean, safe, and cost‐effective alternative to conventional lithium‐ion batteries systems.^[^ ^1^, ^2^, ^3^, ^4^, ^5^, ^6^, ^7^ ^]^ Recent global discussions in academia and industry have focused on finding sustainable and efficient energy storage solutions using Earth‐abundant, non‐toxic, and non‐flammable water‐based systems.^[^ ^3^, ^4^, ^8^, ^9^, ^10^, ^11^ ^]^ The desire to move toward high‐performance aqueous aluminum‐ion batteries (AAIBs) due to its ability to undergo a three‐electron (Al^3+^ or Al(III)) transfer redox reaction, which offers a significantly higher theoretical volumetric capacity of 8046 mAh cm^−3^, four times greater than that of lithium.^[^ ^1^, ^12^, ^13^, ^14^, ^15^, ^16^, ^17^, ^18^, ^19^ ^]^ However, the primary scientific challenge in advancing AAIBs is the direct reversible plating/stripping of the Al metal anode, due to its low deposition potential (−1.667 V vs SHE) and surface passivation.^[^ ^20^, ^21^, ^22^ ^]^ To address these challenges, it is imperative to search for alternative anode materials. Unfortunately, the strong electrostatic interaction between the crystalline lattice of the host electrode and the high charge density of Al^3+^ adversely affects reaction kinetics and reversibility.^[^ ^10^, ^19^, ^22^ ^]^ Therefore, anode materials should possess good electron transport properties, such as high carrier mobility and electronic conductivity, which can facilitate the diffusion of Al^3^⁺ by overcoming the strong electrostatic interaction.^[^ ^1^, ^12^, ^23^ ^]^

In the pursuit of an efficient kinetic promoter, we discovered that novel topological insulators (TIs) could be a compelling option for AAIBs. It is particularly noteworthy that TIs are a new type of quantum substance that behaves as insulators in their interior, but exhibit a gap‐free metallic state on their surface, known as the Dirac cone surface band structure.^[^ ^24^, ^25^, ^26^ ^]^ This suggests that electrons can freely transfer exclusively along the 2D surface of the material, with extraordinary symmetry‐protected topological order.^[^ ^25^, ^26^, ^27^, ^28^ ^]^ These features indicate that reducing the thickness of TIs could boost electrical conductivity by maximizing the exposed surface. However, the influence of the thickness of TIs on charge storage during electrochemical responses has rarely been investigated.

In this work, Bi_2_Te_3_ is chosen to explore the effectiveness of TIs as an anode for AAIBs. Various nanostructures of Bi_2_Te_3_, ranging from low to high specific surface area, are synthesized via solvothermal methods. We conclusively demonstrate that the thinnest Bi_2_Te_3_ nanostructures possess superior charge storage capabilities compared to their bulk counterparts, owing to their larger exposed surface states, which are inherently conductive. The charge storage mechanism of Bi_2_Te_3_ is elucidated through a series of experimental and computational studies. In aqueous aluminum‐based electrolytes, while hydrated Al^3+^ ions are serving as the primary redox charge carriers, the role of protons (H⁺) should not be overlooked due to their small size and light weight compared to other cations.^[^ ^29^, ^30^, ^31^ ^]^ However, bare H⁺ is not typically involved as a charge carrier in aqueous media due to the spontaneous formation of hydronium ions (H_3_O^+^). The dehydration energy of H_3_O^+^ is very high (11.66 eV), which hinders the desolvation process (H_3_O^+^ → H⁺ + H_2_O).^[^ ^29^, ^32^ ^]^ As a consequence, H_3_O^+^ serves as the dominant charge carrier in aqueous electrolytes, rather than bare H⁺.^[^ ^13^, ^30^, ^33^ ^]^ Through Density Functional Theory (DFT) calculations, we reveal that the charge storage mechanism of Bi_2_Te_3_ is primarily governed by the intercalation and deintercalation of H_3_O^+^ ions into its crystal lattice and strong adsorption of hydrated Al^3+^ on its surface. Therefore, developing electrode material with a high surface adsorption capacity is expected to significantly enhance the performance of AAIBs.

To ensure effective surface adsorption, we decorate the optimized Bi_2_Te_3_ nanostructure with polypyrrole (PPy) nanotubes, which have a conjugated backbone capable of interacting efficiently with ions through electrostatic and π‐π interactions.^[^ ^34^ ^]^ Consequently, the Bi_2_Te_3_@PPy anode demonstrates enhanced charge storage characteristics compared to pristine Bi_2_Te_3_ in aluminum‐based electrolytes. DFT calculations further confirm that PPy exhibits a stronger affinity toward Al^3+^ than Bi_2_Te_3_, indicating that ions are more easily captured by Bi_2_Te_3_@PPy. Furthermore, the assembled Bi_2_Te_3_@PPy//LiMnPO_4_ AAIBs exhibited a wide voltage window of 1.8 V, achieving a high energy density of 89 Wh kg^−1^ at a power density of 355 W kg^−1^, with 100% capacity retention over 1000 cycles. We believe the fundamental insights gained from this study will hopefully inspire the further development of TI‐based materials as high‐performance anodes for AAIBs.

## 2 Results and Discussion

### 2.1 Physical Characterization

**Figure 1:**
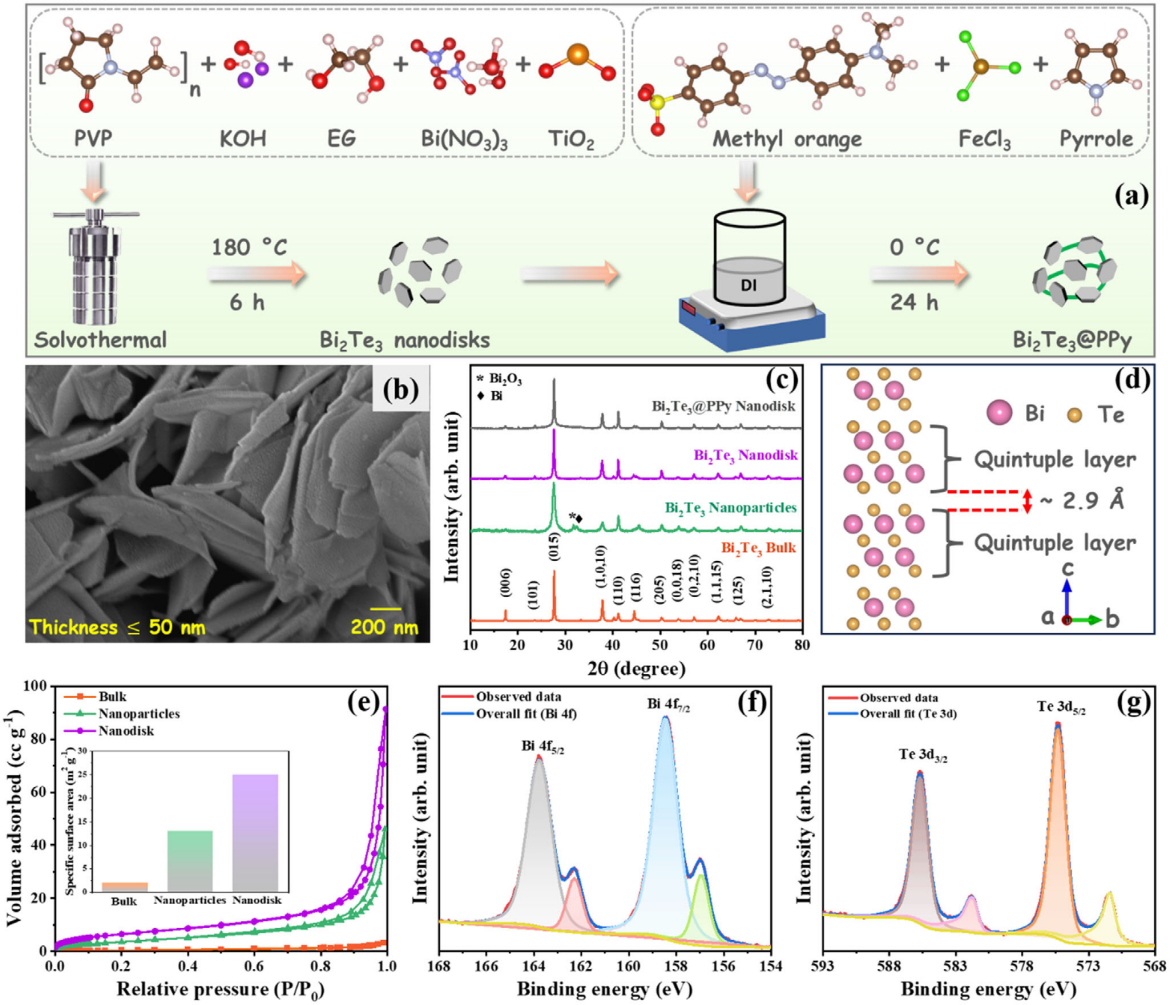
Synthesis procedure and physicochemical characterization results. a) Schematic of synthesis procedure of Bi_2_Te_3_ and Bi_2_Te_3_@PPy nanodisks; b) SEM image of Bi_2_Te_3_ nanodisk; c) XRD patterns of bulk, nanoparticles, nanodisks of Bi_2_Te_3_ and Bi_2_Te_3_@PPy nanodisk; d) crystal structure of Bi_2_Te_3_ (side view of the hexagonal unit cell including quintuple layers); e) N_2_ adsorption‐desorption isotherm of bulk, nanoparticles, and nanodisks of Bi_2_Te_3_ (Inset Figure: comparison of specific surface area); high resolution XPS spectra of f) Bi 4f and g) Te 3d in the Bi_2_Te_3_ nanodisk.

A simple one‐step solvothermal approach, utilizing different solvents, was employed to fabricate various nanostructures of Bi_2_Te_3_. Bi_2_Te_3_@PPy nanodisks were obtained through the self‐polymerization of pyrrole, which served to connect the Bi_2_Te_3_ particles, as illustrated in **Figure** **1**a. The details of these synthesis procedures are described in the Experimental Section (Supporting Information). Scanning electron microscopy (SEM) images, illustrated in Figure S3a—c (Supporting Information) and Figure 1b, confirm that Bi_2_Te_3_ particles synthesized using ethanol and ethylene glycol as solvents form distinct nanostructures, i.e., agglomerated nanoparticles and hexagonal nanodisks geometries, respectively. For comparison, the SEM micrograph of commercial bulk Bi_2_Te_3_ is provided in Figure S3d (Supporting Information). Additionally, Figure S4a (Supporting Information) shows an SEM image of PPy obtained from the unsupported polymerization of pyrrole, while Figure S4b (Supporting Information) presents an SEM image of the Bi_2_Te_3_@PPy nanodisks. The SEM image reveals that Bi_2_Te_3_@PPy is composed of Bi_2_Te_3_ nanodisks interconnected by PPy fibers, which may enhance electron transfer through the network and improve the surface adsorption capacity of Bi_2_Te_3_ during electrochemical response.

The X‐ray diffraction (XRD) patterns of bulk Bi_2_Te_3_ and its nanostructures reveal the distinctive peaks corresponding to the hexagonal Bi_2_Te_3_ phase (PDF#89‐2009, Figure 1c). The (006) peak intensity of Bi_2_Te_3_ nanodisks is relatively weaker than that of bulk, and is even weaker for nanoparticles, reflecting the random orientation of particles having spherical and disk‐shaped geometries with predominant {0001} facets.^[^ ^35^ ^]^ Bi_2_Te_3_ nanoparticles synthesized via ethanol exhibit traces of Bi and Bi_2_O_3_ impurities, which could affect their electrochemical properties and will be discussed in detail in the electrochemical characterization section. The XRD patterns of Bi_2_Te_3_@PPy also exhibit the characteristic peaks of the hexagonal Bi_2_Te_3_ phase, with no additional peaks, indicating the amorphous nature of PPy. Moreover, the absence of shifts in Bi_2_Te_3_‐related XRD peaks confirms that the PPy coating does not alter the crystal structure of Bi_2_Te_3_. Theoretically, the crystal structure of Bi_2_Te_3_ consists of quintuple layers connected by noncovalent van der Waals forces, with each quintuple layer terminated by tellurium atoms, resulting in a (111) cleavage plane that exhibits a hexagonal structure. Figure 1d illustrates the hexagonal unit cell of Bi_2_Te_3_, with the van der Waals gap of ∼ 2.9 Å between two quintuple layers.^[^ ^36^, ^37^ ^]^ This spacing may be advantageous for the diffusion and intercalation/deintercalation of ions from the electrolyte.

N_2_ adsorption/desorption isotherms were used to analyze the surface area and pore size distribution of the synthesized Bi_2_Te_3_ powders (Figure 1e; Figure S5 and Table S1, Supporting Information). As calculated by the Brunauer–Emmett–Teller method, Bi_2_Te_3_ nanodisk exhibits a higher specific surface area of 25 m^2^ g^−1^, associated with the presence of macropores with a net pore volume of 141 mm^3^ g^−1^. In contrast, Bi_2_Te_3_ nanoparticles and bulk Bi_2_Te_3_ display specific surface areas of only 13 m^2^ g^−1^, and 2 m^2^ g^−1^, respectively, with net pore volumes of 71 mm^3^ g^−1^ and 5 mm^3^ g^−1^. These differences in specific surface area and pore volume are expected to significantly influence the ion storage behavior of Bi_2_Te_3_, attributed to the presence of the gap‐free metallic state on the surface of the TI.

To elucidate the elemental composition and valence state of elements in Bi_2_Te_3_ nanodiscs, X‐ray photoelectron spectroscopy (XPS) analysis was conducted. The XPS survey spectrum in Figure S6 (Supporting Information) indicates that the Bi_2_Te_3_ nanodisks are predominantly composed of Bi and Te, as evidenced by their distinct characteristic peaks.^[^ ^38^, ^39^ ^]^ In the high‐resolution XPS spectrum of Bi 4f (Figure 1f), the peaks at 158.5 and 163.7 eV correspond to Bi 4f_7/2_ and Bi 4f_5/2_ in Bi_2_Te_3_, while the peaks at 156.9 and 162.3 eV are attributed to surface oxidation of Bi_2_Te_3_.^[^ ^37^, ^39^, ^40^ ^]^ For the Te 3d XPS spectra (Figure 1g), the peaks at 575.3 and 585.7 eV represent Te 3d_5/2_ and Te 3d_3/2_ in Bi_2_Te_3_, while the peaks at 571.4 and 581.8 eV are consistent with the oxidized layer of Bi_2_Te_3_.^[^ ^37^, ^39^, ^40^ ^]^ This characterization analysis provides clear evidence that the Bi_2_Te_3_ nanodisks were successfully prepared.

### 2.2 Electrochemical Study I – Comparison of Al^3+^ Storage Performance between Bulk and Nanostructured Bi_2_Te_3_

**Figure 2:**
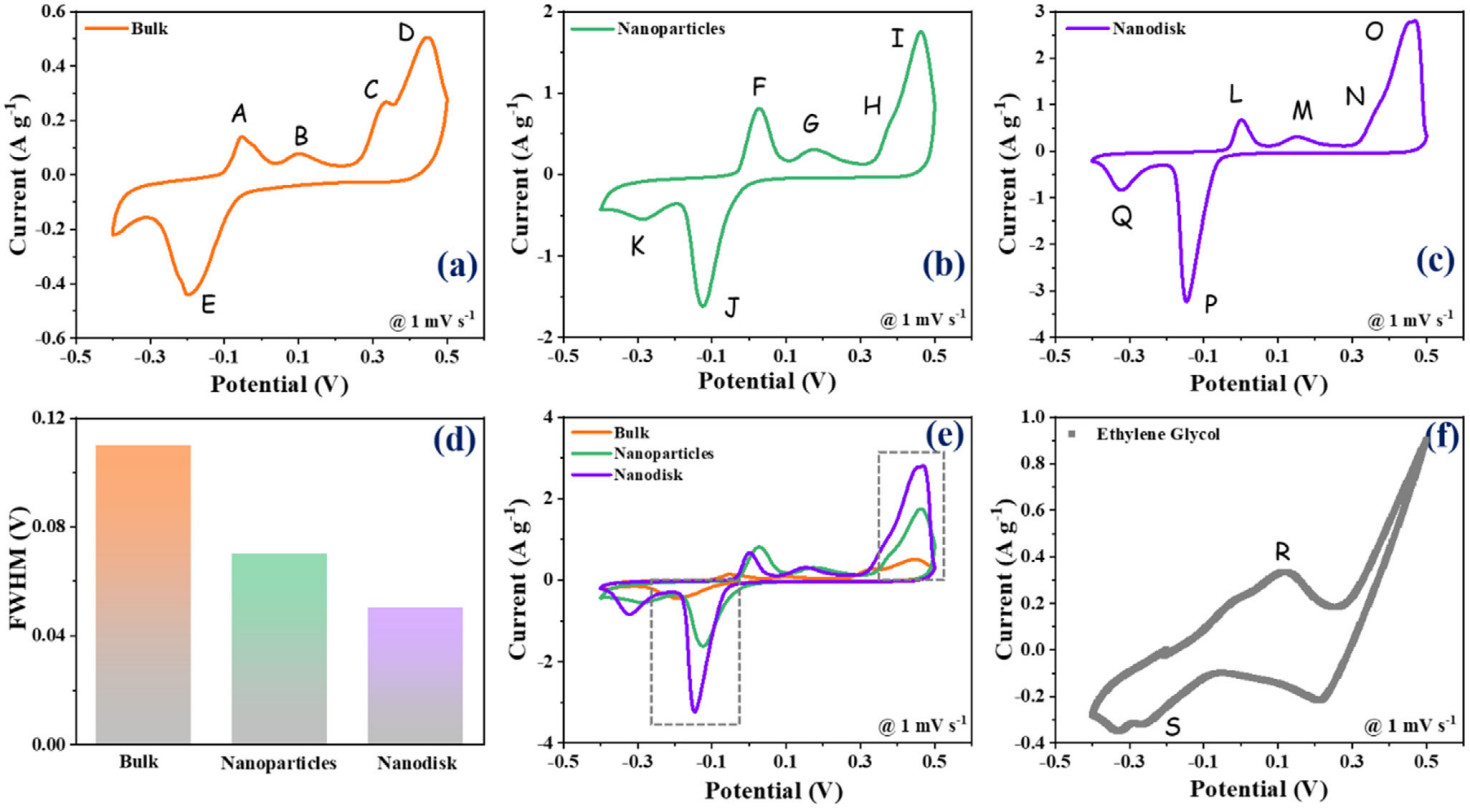
Electrochemical analysis of bulk and nanostructures of Bi_2_Te_3_ from cyclic‐voltammetry (CV) profile. CV profiles of a) bulk Bi_2_Te_3_, b) Bi_2_Te_3_ nanoparticles, and c) Bi_2_Te_3_ nanodisks at 1 mV s^−1^; d) comparison of the FWHM of the reduction peak; e) comparison of CV profiles at 1 mV s^−1^; and f) CV profile of Bi_2_Te_3_ nanodisks in ethylene glycol solvent.

The electrochemical behavior of bulk Bi_2_Te_3_ and the synthesized Bi_2_Te_3_ nanostructures was initially investigated using cyclic voltammetry (CV), performed at a scan rate 1 mV s^−1^ within a voltage window of −0.4 to 0.5 V, as illustrated in **Figure** **2**a–c. All the CV profiles exhibited similar shapes, with the primary distinction being the intensity of the oxidation/reduction peak. It is noteworthy that, while the number of oxidation peaks remains the same across all profiles, the bulk sample displays only one broad reduction peak (“E”), in contrast to the nanoparticles and nanodisks, which show two reduction peaks separately (“K”, “J” for nanoparticles and “P”, “Q” for nanodisks). This broad reduction peak in the bulk Bi_2_Te_3_ might result from the overlapping of two peaks that are individually distinguishable in the nanoparticle and nanodisk samples. The oxidation/reduction peak arises in the CV profile either from ion intercalation/deintercalation into/from the host electrode into the electrolyte or from the adsorption of electrolyte ions on the electrode surface. Sharper oxidation/reduction peaks (“D”, “I”, “O”/“E”, “J”, “P”) often indicate a more prominent or well‐defined ion intercalation/deintercalation process. The extent of peak broadening was determined using the full width at half maximum (FWHM), as illustrated in Figure 2d. The higher FWHM value of bulk Bi_2_Te_3_ reflects slower kinetics with fewer accessible active sites.^[^ ^41^, ^42^, ^43^ ^]^ Agglomerated nanoparticles, with a higher surface area than the bulk, show a lower FWHM value, suggesting improved but still limited ion diffusion due to particle agglomeration. Thin nanodisks exhibit the lowest FWHM value, suggesting that the ion deintercalation is occurring in a more reversible and rapid manner, with less diffusion limitation, driven by their large surface area and short diffusion pathways, which allow for rapid and uniform ion insertion. To highlight the distinctions between the CV profiles of these three morphologies, the profiles were also compared at a fixed scan rate, where the nanostructured morphologies exhibited noticeably stronger oxidation and reduction peak intensities than the bulk Bi_2_Te_3_ (as shown in Figure 2e). This finding further confirmed the slower ion diffusion in bulk Bi_2_Te_3_, where the insulating bulk limits the contribution of conductive surface states. In contrast, the thin nanodisks, with their larger specific surface area, promote the activity of topologically protected surface states, resulting in more favored interaction with the electrolyte. Thus, the CV profile analysis emphasizes the critical role of topological surface states in governing the ion diffusion kinetics in the Bi_2_Te_3_ anode. The CV profiles of bulk and nanostructured Bi_2_Te_3_ at various scan rates are shown in Figure S7 (Supporting Information).

In AAIBs, Al^3+^ can be stored either through the deposition/dissolution of inorganic salts, such as hydrated aluminum‐ion ([Al(H_2_O)_6_]^3+^), on the electrode surface, or via the intercalation/deintercalation of Al^3^⁺ into the crystal lattice. In an aqueous AlCl_3_ electrolyte, compared to Al^3+^, protons are also readily dissolved and intercalated.^[^ ^13^ ^]^ Since protons have smaller sizes and weaker electrostatic interactions with the crystal lattice than Al^3+^, so the intercalation/deintercalation of protons is also expected with the addition of primary charge carriers Al^3+^. Hence, a pertinent question is whether the observed activity of the Bi_2_Te_3_ anode in the CV profile is predominantly driven by Al^3+^ intercalation or proton intercalation. To verify this, we use ethylene glycol (EG) organic solvent to prepare the AlCl_3_ electrolyte instead of water, as EG is suitable as an electrolyte solvent and inhibits proton ionization.^[^ ^44^ ^]^ The CV profile of Bi_2_Te_3_ nanodisks in EG solution is shown in Figure 2f. The Bi_2_Te_3_ nanodisk anode exhibited broad oxidation and reduction peaks in the potential window of ≈0 to 0.2 V (“R”) and ≈−0.2 to −0.4 V (“S”), respectively. On the other hand, the CV profile of the Bi_2_Te_3_ nanodisk in an aqueous solution also showed oxidation peaks in the same potential window of ≈0 to 0.2 V. Therefore, the presence of this peak may correlate with the intercalation process of complex Al^3+^ ions. Similarly, the broad peak in EG solution, in the range of ≈−0.2 to −0.4 V, is related to the peak located in the aqueous solution at ≈−0.32 V, which can be attributed to the deintercalation process of Al^3+^ complex. Now, the most intense oxidation and reduction peaks in the aqueous solution appeared at ≈0.46 and ≈−0.14 V, respectively, which do not match any of the peaks in an organic solvent. This suggests that these intense peaks in the aqueous solution may be related to the intercalation/deintercalation process of protons. This finding highlights the critical role of proton intercalation/deintercalation in the performance of the Bi_2_Te_3_ anode for AAIBs. It is worth noting that the magnitude of oxidation/reduction peak currents in the EG solution is significantly lower than in the aqueous solution. This difference can be attributed to the lower ionic conductivity of the organic solvent compared to the aqueous medium.^[^ ^45^ ^]^ Consequently, this effect results in a higher equivalent series resistance (ESR) value of ≈223 Ω at the electrode‐electrolyte interface, as illustrated in Figure S8 (Supporting Information). Additionally, when AlCl_3_ is dissolved in ethylene glycol, it generally forms complexes with ethylene glycol molecules rather than simple hydrated ions as in water, and the ionic or molecular size of these aluminum‐glycol complexes is generally larger than that of the hydrated aluminum ion. Since EG is a protic solvent, we also evaluated the electrochemical performance of Bi_2_Te_3_ nanodisks in various aprotic solvents, as discussed in Figure S9 (Supporting Information).

**Figure 3:**
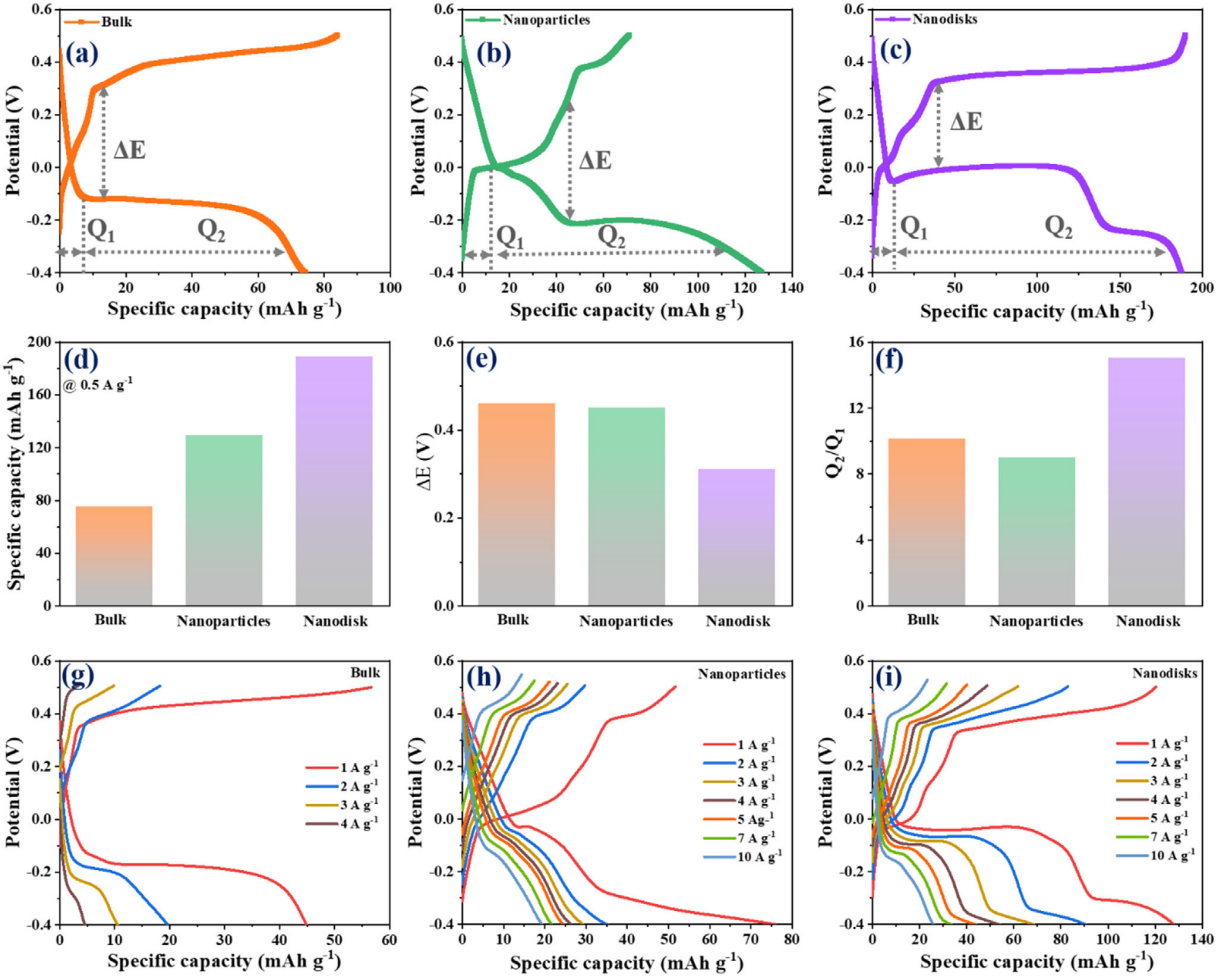
Electrochemical analysis of bulk and nanostructures of Bi_2_Te_3_ from Galvanostatic charge‐discharge (GCD) profile. GCD profiles of a) bulk Bi_2_Te_3_, b) Bi_2_Te_3_ nanoparticles, and c) Bi_2_Te_3_ nanodisk at 0.5 A g^−1^; d) comparison of specific capacity, e) statistics of charge potential barrier, and f) statistics of capacity from plateau for bulk, nanoparticles, and nanodisk of Bi_2_Te_3_; rate capability performance of g) bulk Bi_2_Te_3_, h) Bi_2_Te_3_ nanoparticles, and i) Bi_2_Te_3_ nanodisk at various current densities.

The galvanostatic charge‐discharge (GCD) profiles of bulk and nanostructured Bi_2_Te_3_ at a current density of 0.5 A g^−1^ are shown in **Figure** **3**a–c. A distinct pair of flat plateaus can be observed, which is consistent with the CV curves. It is important to highlight that the diminished discharge plateau was observed for the nanoparticles synthesized using ethanol. This is likely due to the formation of trace amounts of Bi_2_O_3_, as discussed in the material characterization section. The presence of Bi_2_O_3_ as a secondary phase can induce surface defects by disrupting the local crystallinity and introducing electronic inhomogeneity in the Bi_2_Te_3_ crystal structure.^[^ ^46^, ^47^, ^48^ ^]^ These defects can act as trapping sites for charge carriers and hinder ion diffusion pathways,^[^ ^49^ ^]^ which may enable irreversible or parasitic redox processes during discharging. Moreover, the presence of oxidized spices in the topological material leads to the deterioration of topological surface states,^[^ ^47^ ^]^ which in turn alters the electrochemical behavior of the nanoparticles by disrupting the smooth ion intercalation/deintercalation processes that contribute to the formation of a clear charge–discharge plateau in Bi_2_Te_3_. Consequently, the analysis of the GCD profile highlights that achieving a prominent and facile intercalation/deintercalation of ions requires the Bi_2_Te_3_ material to be in its pure phase, free from a trace amount of bismuth metal or oxide impurities. While Bi_2_Te_3_ nanoparticles exhibit some surface impurities, the transition from bulk Bi_2_Te_3_ to agglomerated nanoparticles results in enhanced specific capacity, primarily due to the presence of more topological surface states. Among the three different Bi_2_Te_3_ materials, the nanodisk morphology exhibits a significantly higher specific capacity of 189 mAh g^−1^, compared to the much lower values of 129 mAh g^−1^ for agglomerated nanoparticles and 75 mAh g^−1^ for bulk Bi_2_Te_3_ at the same current rate, as depicted in Figure 3d. The higher electrochemical performance of the nanodisks can be attributed to the polarization effect of the electrode. The potential difference between the charging and discharging plateaus (ΔE) typically indicates the electrochemical polarization effect of the electrode. The nanodisk Bi_2_Te_3_ exhibits a lower ΔE, as shown in Figure 3e, indicating reduced electrochemical polarization, which benefits from the highly exposed topological quantum surface states. The contribution of capacity from the discharging plateau ($\frac{Q_{2}}{Q_{1}}=\frac{capacity\;from\;discharging\;plateau}{capacity\;other\;than\;plateau}$) is also a key descriptor for the discharge process.^[^ ^50^, ^51^ ^]^ This contribution is higher for nanodisks compared to bulk and nanoparticles, indicating improved charge storage performance (Figure 3f). Figure 3g–i presents detailed GCD profiles of electrodes based on bulk Bi_2_Te_3_, Bi_2_Te_3_ nanoparticles, and Bi_2_Te_3_ nanodisks at various current rates. Due to the long ion diffusion distance, a significant decrease in specific capacity is observed with increasing current density for the bulk Bi_2_Te_3_, and at 4 A g^−1^, it shows almost negligible specific capacity. In contrast, due to the greater abundance of topological surface states, Bi_2_Te_3_ nanoparticles exhibit better rate capability than the bulk material, with a specific capacity of ≈19 mAh g^−1^ at a high current rate of 10 A g^−1^. However, at lower current densities, the discharge capacity is slightly higher than the charge capacity, possibly due to the partial irreversibility of surface‐related processes arising from the presence of a secondary Bi_2_O_3_ phase. Like nanoparticles, Bi_2_Te_3_ nanodisk also demonstrates impressive rate performance, maintaining a prominent discharge plateau at each current rate, with corresponding discharge capacities of 129, 90, 69, 54, 44, 33, and 27 mAh g^−1^ at 1, 2, 3, 4, 5, 7, and 10 A g^−1^, respectively. Nowadays, the majority of electrode materials reported in the literature lack a distinct discharge plateau.^[^ ^13^, ^14^, ^15^, ^52^, ^53^, ^54^, ^55^, ^56^ ^]^ However, the Bi_2_Te_3_ nanodisk presented here demonstrates a prominent discharge plateau, even at higher current rates (Figure 3i). The equivalent series resistance (ESR) of the Bi_2_Te_3_ nanodisk electrode was also lower than that of its bulk and nanoparticle counterparts (Figure S10 and Table S2, Supporting Information). Hence, we conclude that the superior electrochemical behavior observed in thinner Bi_2_Te_3_ nanostructures can be directly attributed to the greater influence of topological surface states, which become more prominent at reduced thicknesses. While it is true that nanostructuring can enhance the performance of many conventional materials by increasing surface area and reducing ion diffusion paths,^[^ ^7^ ^]^ TIs provide an additional mechanism for performance enhancement.^[^ ^24^, ^26^, ^57^ ^]^ Specifically, their gapless, high‐mobility surface states are inherently active in charge transport and surface adsorption, quantum features absent in traditional electrode materials.^[^ ^26^, ^58^, ^59^ ^]^

### 2.3 Electrochemical Study II – Proton and Other Metal Cation Intercalation

The influence of different cation species in Bi_2_Te_3_ nanodisks was investigated using electrolytes containing Na^+^, K^+^, Ca^2+^, and Mg^2+^ ions. Figure S11a—d (Supporting Information) displays CV curves at 1 mV s^−1^ in NaCl, KCl, CaCl_2_, and MgCl_2_ electrolytes. Unlike the distinct redox peaks observed in the AlCl_3_ electrolyte, the other electrolytes exhibited no prominent redox activity, with the cyclic voltammetry current being ten orders of magnitude lower than that achieved in the AlCl_3_ electrolyte. Consequently, GCD profiles (Figure S11e–h, Supporting Information) revealed negligible capacity in these electrolytes. Figure S11i (Supporting Information) compares the specific capacities of Bi_2_Te_3_ nanodisks in different electrolytes, highlighting an optimal capacity of 189 mAh g^−1^ uniquely achieved in the AlCl_3_ electrolyte.

It is worth noting that cations are present in aqueous solution in their hydrated form, and the order of the hydrated ionic radii is Al^3+^ (4.75 Å) > Mg^2+^ (4.28 Å) > Ca^2+^ (4.12 Å) > Na^+^ (3.58 Å) > K^+^ (3.31 Å).^[^ ^10^ ^]^ Aluminum has the highest valency state of +3 among these ions and hence, it strongly attracts water molecules, forming a large hydration shell. Thus, a pertinent question arises: despite the larger hydrated ionic radius of Al^3+^, why are the redox peaks in AlCl_3_ electrolyte more prominent compared to NaCl, KCl, MgCl_2_, and CaCl_2_ electrolyte? To address this question, we analyze the differences in the compositions of these electrolytes. When aluminum chloride (AlCl_3_) dissolves in water, the Al^3+^ ions become hydrated, forming the hexaaqua complex [Al(H_2_O)_6_]^3+^. Due to the high charge density of the aluminum ion, the water molecules in the complex become polarized. This can lead to hydrolysis, where a proton (H^+^) is released from the water molecules attached to Al^3+^, creating hydronium ions (H_3_O^+^) in the solution. The reaction is as follows:

(1) $${\left[Al{\left(H_{2}O\right)}_{6}\right]}^{3+}\to {\left[Al{\left(H_{2}O\right)}_{5}\left(OH\right)\right]}^{2+}+H^{+}$$

(2) $$H^{+}+H_{2}O\to H_{3}O^{+}$$

The solvation energy of a proton is very low, estimated to be −11.38 (±0.07) eV, which accelerates the formation of H_3_O^+^.^[^ ^29^, ^60^, ^61^ ^]^ Therefore, naked H^+^ ions are rarely present in the electrolyte. Consequently, in aqueous AlCl_3_ electrolytes, apart from hydrated Al^3+^ serving as the primary redox charge carriers, the significance of hydronium ions (H_3_O^+^) should not be disregarded. Protons formed by H_3_O^+^ desolvation possess a smaller ion radius, lower ion mass, and exhibit ultrafast diffusion kinetics, rendering it an ideal charge carrier. In contrast, the concentration of H_3_O^+^ or H^+^ is negligible in neutral solutions of NaCl, KCl, CaCl_2_, and MgCl_2_ electrolytes under standard conditions, as shown in Table S3 (Supporting Information). This confirms that a significant contribution to capacity in AlCl_3_ electrolyte is associated with the intercalation/deintercalation of H_3_O^+^ ion rather than the intercalation/deintercalation of Al^3+^ ion. We also monitored the pH variation of the 0.5 AlCl_3_ electrolyte during the charge/discharge process to demonstrate the proton involvement in the storage mechanism (see Supporting Information).

To further assess the activity of proton intercalation, electrochemical measurements were also carried out in 0.5 m HCl and H_2_SO_4_ electrolytes. The CV profile (Figure S12a,b, Supporting Information) revealed the presence of intense oxidation and reduction peaks. Although the peak potential due to hydronium ion intercalation matches exactly, the peak potential for the hydronium ion deintercalation process does not precisely match the peaks observed in the AlCl_3_ electrolyte. These differences arise from variations in H_3_O^+^ concentration and counterion effects. In both the HCl and H_2_SO_4_ electrolytes, the H_3_O^+^ concentration is significantly higher due to complete dissociation, whereas in AlCl_3_, H_3_O^+^ is generated through the hydrolysis of Al^3+^, resulting in a lower effective H_3_O^+^ concentration (Table S3, Supporting Information). This distinction in proton availability directly impacts the electrode intercalation process and the observed peak potentials. Furthermore, the counter‐ions present in the electrolyte play a critical role in modifying the electrochemical environment. In H_2_SO_4_, the counter‐ions are HSO_4_^−^ and SO_4_^2−^ from the dissociation of H_2_SO_4_, while in HCl, the counterion is a single Cl^−^, which may interact differently with the electrode material compared to the three Cl^−^ ions in AlCl_3_. These differences, both in proton concentration and counterion behavior, influence the ion intercalation dynamics and contribute to the observed variations in redox peak potentials. However, in both the HCl and H_2_SO_4_ electrolytes, a substantial decrease in peak intensity was observed in the subsequent cycle, as shown in Figure S12a,b (Supporting Information), likely due to the degradation of the electrode material caused by the strong acidic environment.^[^ ^52^, ^62^ ^]^ Despite the active role of protons, the first discharge capacities in H_2_SO_4_ and HCl electrolytes are only 47 and 61 mAh g^−1^, respectively (Figure S12c,d, Supporting Information). These values are significantly lower than the achieved capacity (189 mAh g^−1^) in AlCl_3_ electrolyte, emphasizing the combined contribution of aluminum and hydronium ions to the performance of Bi_2_Te_3_ nanodisks. Figure S13 (Supporting Information) demonstrates how different cations in various electrolytes influence the charge storage capability of Bi_2_Te_3_ nanodisks.

To further understand the charge storage mechanism in the AlCl_3_ electrolyte, we also evaluated the electrochemical performance of Bi_2_Te_3_ nanodisks in a diluted HCl electrolyte (≈0.003 m). The pH of the ≈0.003 m HCl solution was similar to that of the 0.5 m AlCl_3_ solution, i.e., ≈3.9 at ≈21 °C. The corresponding CV and GCD profiles in the diluted HCl electrolyte are presented in Figure S14 (Supporting Information). According to the CV profile, the positions of the oxidation and reduction peaks in the diluted HCl electrolyte closely matched with the most intense peaks observed in the 0.5 m AlCl_3_ electrolyte. This further confirms the dominant role of H_3_O^+^ ions in the AlCl_3_ electrolyte. However, the GCD profile in the diluted HCl electrolyte exhibits a negligible discharge time, suggesting that protons generated solely from the diluted HCl are insufficient to sustain a high capacity. Although the discharge time was negligible, a stable charge–discharge plateau was observed in the diluted HCl electrolyte, similar to that in the 0.5 m AlCl_3_ electrolyte. As >80% of the capacity contribution in the 0.5 m AlCl_3_ electrolyte originates from this plateau region, this may again suggest that the appearance of the plateau in the GCD profile of Bi_2_Te_3_ is likely attributable to the intercalation and deintercalation of H_3_O^+^ ions. Thus, we can conclude that in the AlCl_3_ electrolyte, hydrated Al^3+^ ions strongly adsorb onto the host electrode surface during charging, releasing protons through hydrolysis. These protons actively participate in the intercalation process, resulting in a more stable plateau and a significantly higher discharge capacity. Therefore, although the proton concentration is nominally equivalent in both the 0.5 m AlCl_3_ and the ≈0.003 m HCl electrolytes, the discharge capacity is substantially lower in the latter. This result highlights that not only the proton concentration but also the dynamic generation of protons via hydrolysis of hydrated Al^3+^ ions play a critical role in stabilizing the electrochemical performance.

### 2.4 Charge Storage Mechanism and DFT Calculations of Ion Intercalation and Diffusion

**Figure 4:**
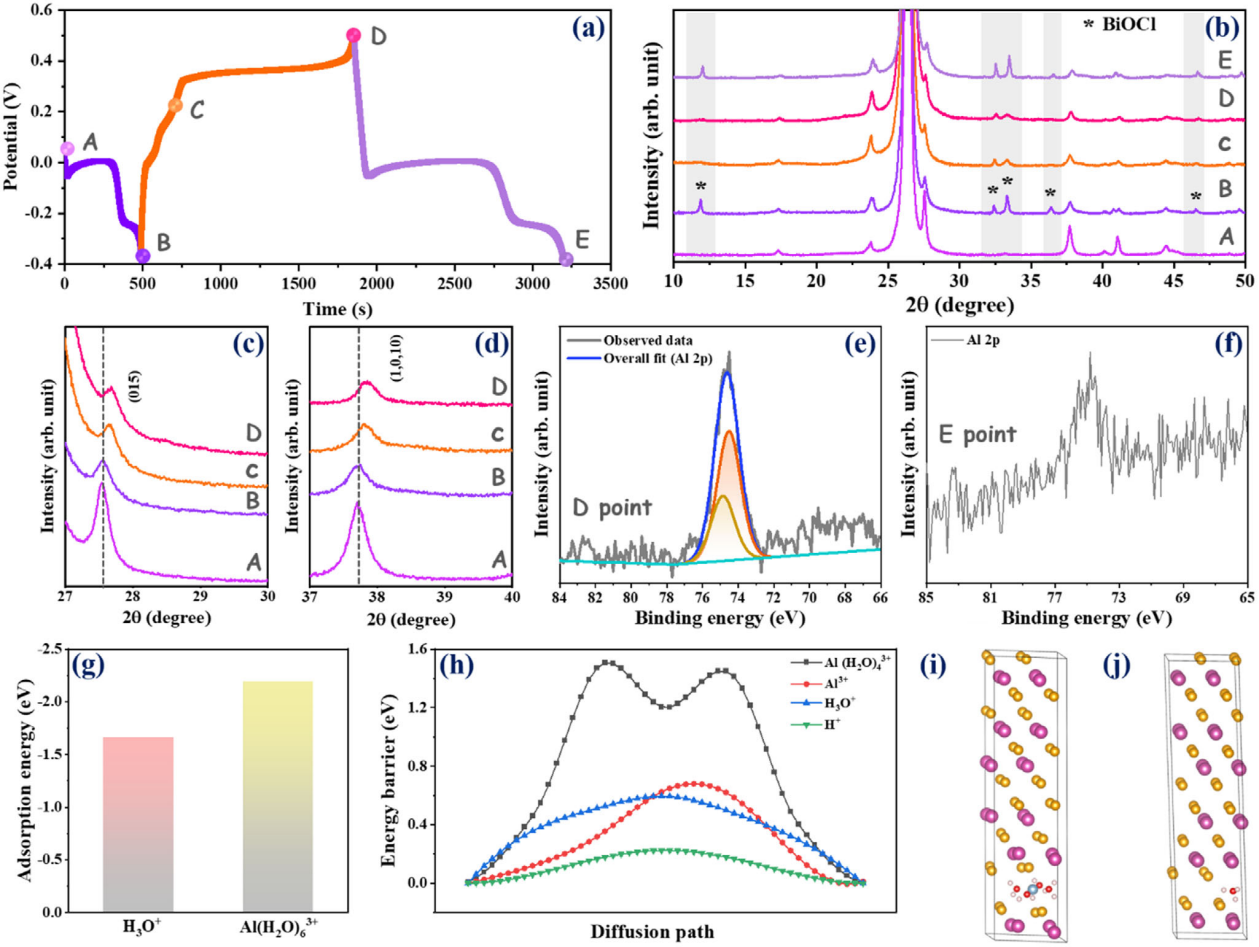
Ex situ characterizations and DFT calculations of ion intercalation and diffusion in Bi_2_Te_3_ nanodisk: a) Charge and discharge states labeled from A to E with corresponding charge–discharge profile, b) Ex situ XRD pattern, and change of the interlayer spacing due to the intercalation/deintercalation of ions for (c) (015), and (d) (1,0,10) plane; Ex situ XPS spectra of aluminum in e) charged (point D) and f) discharged state (point E); g) comparison of adsorption energies for H_3_O^+^ and Al(H_2_O)_6_^3+^; h) the diffusion barriers for diffusion of ionic species through van der Waals gap of Bi_2_Te_3_; equilibrium position of i) Al(H_2_O)_4_^3+^ and j) H_3_O^+^ intercalated into Bi_2_Te_3_ (along c‐axis).

To investigate the influence of ion intercalation/deintercalation on the structural evolution of the Bi_2_Te_3_ electrode, we collected XRD patterns at different charging and discharging states (points A to E in **Figure** **4**a), as shown in Figure 4b. Point A corresponds to the XRD pattern of the pristine Bi_2_Te_3_ electrode, where the broad peak observed at 26.3° is attributed to the graphite sheet current collector. After discharging to −0.4 V (point B), a few new peaks appeared in the XRD pattern at 11.8°, 32.3°, 33.2°, and 36.3°, corresponding to the formation of the BiOCl phase (JCPDS 06–0249). During the subsequent charging cycle (points C and D), the intensity of these diffraction peaks decreased significantly, indicating a quasi‐reversible process. In the following discharging cycle (point E), the intensity of the BiOCl phase became prominent again. This suggests that during discharging, chloride ions are adsorbed from the electrolyte, leading to the formation of the bismuth oxychloride phase, i.e., BiOCl. In addition to the appearance of new peaks, it is noteworthy that the d‐spacing of all Bi_2_Te_3_ planes changes when moving from the discharging to charging states (points B to C and D). These changes in d‐spacing are attributed to the increased electrostatic interactions between the intercalated ions and the layers of Bi_2_Te_3_. The changes in d‐spacing due to the intercalation of ions for the (015), (1,0,10), (006), and (101) planes of Bi_2_Te_3_ are shown in Figure 4c,d and Figure S15 (Supporting Information). The ex situ XPS spectra are also collected as shown in Figure 4e,f. Since XPS is a surface‐sensitive technique, it provides information only about the surface state of the electrode material. In the fully charged electrode (Figure 4e), intense aluminum spectra are present, confirming the absorption of aluminum ions from the electrolyte. In contrast, the percentage of absorbed aluminum is negligible in the discharged state of the electrode, as shown in Figure 4f. Therefore, ex‐situ studies reveal that, in addition to ion adsorption, the interlayer spacing of Bi_2_Te_3_ undergoes only a negligible change due to ion intercalation. If aluminum or hydrated aluminum ions were inserted, a larger variation in the interlayer spacing occurs due to the strong electrostatic interactions arising from their trivalent state (the theoretical calculation is presented in the next paragraph). This further supports the intercalation and deintercalation of the uniquely charged hydronium ion. To further elucidate the role of H_3_O^+^ ions, ex‐situ FTIR measurements were performed in Attenuated Total Reflectance mode, as the intercalation and deintercalation of H_3_O^+^ ions may also influence the O─H stretching band at ≈3650 cm^−1^ in the host electrode material. As shown in Figure S16 (Supporting Information), upon charging, this band became significantly more intense, indicating an increased presence of H_3_O^+^ in the electrode. In the subsequent discharge, the intensity of the O─H band decreased relative to the charged state. This suggests the active involvement of H_3_O^+^ ions during the electrochemical response.

To explore the interaction of various ions with Bi_2_Te_3_, DFT calculations were carried out to determine the adsorption energies of ions and their diffusion through the van der Waals gap of Bi_2_Te_3_. The DFT results indicate that [Al(H_2_O)_6_]^3+^ exhibits a lower adsorption energy (−2.19 eV) to the surface compared to H_3_O^+^ (−1.66 eV), as depicted in Figure 4g. This implies that, thermodynamically, [Al(H_2_O)_6_]^3+^ species adsorb strongly than H_3_O^+^. On the other hand, the energy required for H_3_O^+^ diffusion is significantly lower than that required for [Al(H_2_O)_4_]^3+^ as depicted in Figure 4h. For intercalation and diffusion, we considered [Al(H_2_O)_4_]^3+^ because its flat geometry fits within the layers of Bi_2_Te_3_ in contrast to the octahedral geometry of [Al(H_2_O)_6_]^3+^ (see Supporting Information). Although the hexa‐aqua cluster [Al(H_2_O)_6_]^3+^ is stable, with a dissociation energy of 117.5 kcal mol^−1^ to [Al(H_2_O)_4_]^3+^,^[^ ^63^ ^]^ Al^3+^ in aqueous solutions exhibits a strong tendency toward hydrolysis, leading to the formation of various mono‐ and polynuclear hydroxo complexes.^[^ ^64^ ^]^ The energy cost associated with the partial desolvation of [Al(H_2_O)_6_]^3+^ is provided in the Supplementary Information. We also compared the diffusion barriers of bare H^+^ and Al^3+^ ions with those of H_3_O^+^ (Figure 4h). The results indicate there is an extremely low diffusion barrier for H^+^, while the energy barrier for the diffusion of Al^3+^ is nearly comparable to that of H_3_O^+^. However, the probability of H⁺ and Al^3+^ ions being present in the electrolyte is extremely low compared to H_3_O^+^ and [Al(H_2_O)_6_]^3+^ due to hydrolysis of the electrolyte. Hence, H_3_O^+^ ions will play a dominant role over H⁺ and Al^3^⁺ to store charges in Bi_2_Te_3_ through insertion/deinsertion. We also calculated the relative change in the c‐lattice parameter upon ion intercalation. The variation in the c‐lattice spacing of Bi_2_Te_3_ caused by the intercalation of H_3_O^+^ is lower than that caused by Al^3+^ and [Al(H_2_O)_4_]^3+^. We obtained the values of 0.6%, −2.7%, and 2.5% for relative expansion/contraction due to intercalation of H_3_O^+^, Al^3+^, and [Al(H_2_O)_4_]^3+^, respectively. Hence, this confirmed that intercalation of Al^3+^ and [Al(H_2_O)_4_]^3+^ induces more pronounced changes in the interlayer spacing compared to H_3_O^+^. If [Al(H_2_O)_4_]^3+^ intercalates, it expands the lattice by 0.75 Å at its equilibrium position in the van der Waals gap, whereas Al^3^⁺ shrinks the lattice by 0.81 Å due to strong electrostatic interaction. In contrast, H₃O⁺ increases the spacing by only 0.18 Å in the equilibrium. The modest expansion of the lattice occurs because H_3_O^+^ fits well within the van der Waals gap and polarizes the layers of Bi_2_Te_3_ in the vicinity. The equilibrium positions of intercalated [Al(H_2_O)_4_]^3+^ and H_3_O^+^ into the Bi_2_Te_3_ crystal lattice are shown in Figure 4i‐j. The modest expansion of the lattice occurs because H_3_O^+^ fits well within the van der Waals gap and polarizes the layers of Bi_2_Te_3_ in the vicinity. Therefore, theoretical studies align with experimental observations, confirming that the charge storage mechanism in Bi_2_Te_3_ is mainly controlled by the surface adsorption/desorption of [Al(H_2_O)_6_]^3+^ and the intercalation/deintercalation of H_3_O^+^.

### 2.5 Electrochemical Study III – Charge Storage Performance of Bi_2_Te_3_@polypyrrole

The theoretical investigation, along with the experimental studies, reveals that the charge storage mechanism of Bi_2_Te_3_ is primarily governed by the intercalation and deintercalation of H_3_O^+^ ions and the deposition/dissolution of hydrated aluminum ions on the electrode surface. Therefore, developing electrode material with a high surface adsorption capacity is expected to significantly enhance the performance. PPy is a conductive polymer with a conjugated backbone, allowing it to efficiently interact with ions through electrostatic and π‐π interactions.^[^ ^35^, ^65^ ^]^ This conductive matrix not only improves ion mobility but also facilitates charge transfer across the surface of Bi_2_Te_3_. When combined, PPy provides a supportive scaffold around Bi_2_Te_3_ nanodisks, increasing the effective surface area due to its porous tube‐like structure, which creates more sites for ion adsorption and enhances the interfacial interactions with electrolyte ions. Theoretically, we also observed that the presence of PPy nanotubes enhances the storage capacity due to their strong reactivity toward both [Al(H_2_O)_6_]^3+^ and H_3_O^+^ ions. H_3_O^+^ adsorbs noncovalently with an adsorption energy of −0.22 eV (Figure S17a, Supporting Information). However, a proton transfer from H_3_O^+^ to one of the carbon atoms of PPy leads to a lower energy state (−2.23 eV, Figure S17b, Supporting Information). The initial adsorption of [Al(H_2_O)_6_]^3+^ species is stronger than that of H_3_O^+^, with an adsorption energy of −0.46 eV (Figure S17c, Supporting Information). Similar to H_3_O^+^, [Al(H_2_O)_6_]^3+^ can transfer two protons to PPy, leading to a significant geometric change and the release of water molecules with a corresponding adsorption energy of −3.35 eV (Figure S17d, Supporting Information). Thus, it is plausible that hydrated Al^3+^ ions release some water molecules due to their interaction with PPy, which, in turn, may facilitate their diffusion into Bi_2_Te_3_.

**Figure 5:**
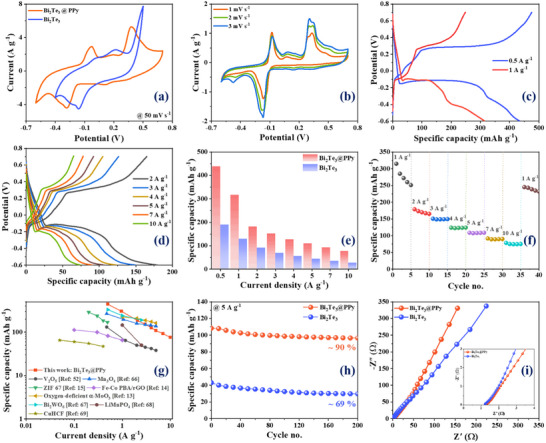
Electrochemical characterization of Bi_2_Te_3_@PPy nanodisk anode. a) Comparison of cyclic voltammetry profiles of Bi_2_Te_3_ nanodisk and Bi_2_Te_3_@PPy nanodisk at a 50 mV s^−1^ scan rate; b) cyclic voltammetry and c,d) charge–discharge profiles of Bi_2_Te_3_@PPy nanodisk at various scan rates and current densities, respectively; e) comparison of specific capacities; f) rate performance of Bi_2_Te_3_@PPy nanodisk; g) rate capability comparison with the reported literature; h) cycling stability; and i) Nyquist plot.

The CV profile of PPy‐decorated Bi_2_Te_3_ nanodisks at a scan rate of 50 mV s^−1^ is depicted in **Figure** **5**a, demonstrating superior electrochemical stability compared to pristine Bi_2_Te_3_. This enhancement is attributed to the PPy coating, which connects the Bi_2_Te_3_ nanodisks and provides a robust mechanical framework for ion and electron migration over a wider electrochemical stability window. CV measurements of Bi_2_Te_3_@PPy at lower scan rates, shown in Figure 5b, exhibit similar redox peaks to those of pristine Bi_2_Te_3_, indicating that the introduction of the PPy coating does not fundamentally change the storage mechanism. On the other hand, a pure PPy electrode exhibits no obvious redox activity in comparison with the composite electrode (Figure S18a, Supporting Information).

A series of GCD tests were performed to systematically assess the electrochemical performance of the composite anode. It can be seen that the Bi_2_Te_3_@PPy anode achieves specific capacities of 438, 317, 180, 152, 126, 109, 92, and 76 mAh g^−1^ at current rates of 0.5, 1, 2, 3, 4, 5, 7, and 10 A g^−1^, respectively (Figure 5c,d). In contrast, the pristine Bi_2_Te_3_ delivers much lower capacities at the same current rates, as compared in Figure 5e. On the other hand, the pristine PPy electrode exhibits a specific capacity of ≈70 mAh g^−1^ at a current rate of 1 A g^−1^ (Figure S18b, Supporting Information). Thus, PPy contributes ≈22% to the total capacity of the Bi_2_Te_3_@PPy composite at 1 A g^−1^. The rate performance of the electrode based on Bi_2_Te_3_@PPy nanodiscs at various rates is presented in Figure 5f. Although a decrease in capacity is observed at a lower current rate of 1 A g^−1^, the retention remains at ≈100% at higher current densities from 3 to 10 A g^−1^. Additionally, when the current density is reduced back to 1 A g^−1^ from 10 A g^−1^, the capacity of Bi_2_Te_3_@PPy fully recovers to 250 mAh g^−1^, with a less noticeable decrement than observed in the initial cycles at 1 A g^−1^. A comparison of the performance with existing literature is presented in Figure 5g ^[^ ^13^, ^14^, ^15^, ^52^, ^66^, ^67^, ^68^, ^69^ ^]^ and Table S4 (Supporting Information), highlighting that the composite material surpasses all previously reported results. Remarkably, even at higher current rates, its performance exceeds that of other materials at much lower current rates. Figure 5h shows the cycling performance of the Bi_2_Te_3_ and Bi_2_Te_3_@PPy anodes. The Bi_2_Te_3_@PPy anode exhibits a capacity retention of 90% after 200 cycles, whereas bare Bi_2_Te_3_ shows a lower retention of 69%. This improvement is attributed to the PPy coating, which sustains the mechanical integrity of the anode during volume variations throughout cycling. Figure 5i displays the Nyquist plots for both the Bi_2_Te_3_ and Bi_2_Te_3_@PPy anodes. The ESR values for both anodes were ≈1.2 Ω. A notable distinction is observed in the lower‐frequency region, which corresponds to the Warburg diffusion process. The increased vertical slope observed in the Bi_2_Te_3_@PPy composite indicates an enhanced surface adsorption capacity, enabling more effective adsorption of ions from the electrolyte.

### 2.6 Device Performance of Aqueous Aluminum‐Ion Batteries (AAIBs)

**Figure 6:**
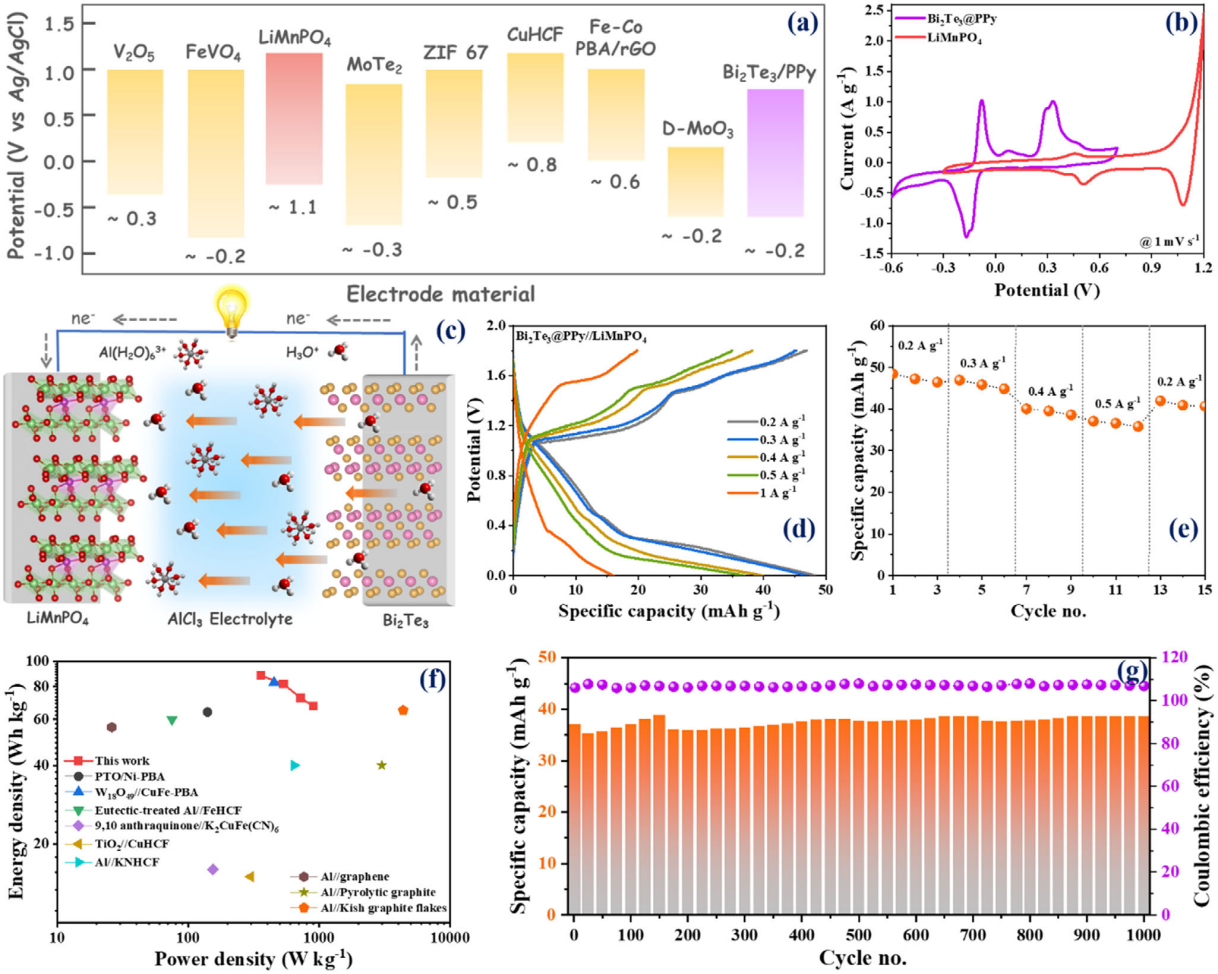
Electrochemical performance of a full cell of AAIBs. a) Operating potential window of the electrode materials for AAIBs reported so far, with values representing the reduction peak potential in AlCl_3_ electrolyte; b) cyclic voltammetry profile representing the overall potential window of AAIBs; c) schematic illustration of the full cell of AAIBs; d) charge‐discharge profile; e) rate capability; f) performance comparison with the reported literature; g) cycling performance and coulombic efficiency at 0.5 A g^−1^ current rate.

**Figure** **6**a illustrates the working potential window of the Bi_2_Te_3_@PPy anode, along with those of some other electrode materials reported for AAIBs.^[^ ^13^, ^14^, ^15^, ^52^, ^56^, ^68^, ^69^, ^70^ ^]^ The Bi_2_Te_3_@PPy exhibits a working potential range from −0.6 to +0.7 V (vs Ag/AgCl), with a reduction peak in the negative potential region (≈−0.2 V), making it a promising anode candidate for AAIBs. Subsequently, LiMnPO_4_ (LMP) was selected as the coupling cathode material in this work due to its reduction peak potential at ≈1.1 V, which is more positive compared to other materials reported so far. Therefore, the wider separation between the reduction peak potentials of the anode and cathode is expected to allow the device to operate within a broader potential window. The CV curves of Bi_2_Te_3_@PPy anode and LMP cathode at the same scan rate are displayed in Figure 6b. Their reduction peaks are concentrated in opposite potential directions, so a cell voltage interval of 1.8 V can be achieved for the assembled full cell. Details of the electrochemical characterization and the synthesis procedure of the LMP cathode are provided in the supplementary information (Figure S19, Supporting Information). A full cell was thus constructed with the schematic two‐electrode configuration illustrated in Figure 6 **c**. The N/P ratio and the details of the charge balance calculation prior to device characterization are provided in the Supporting Information. The GCD profiles of the as‐constructed battery in Figure 6d verified a specific capacity of 49, 47, 40, 37, and 16 mAh g^−1^ at 0.2, 0.3, 0.4, 0.5, and 1 A g^−1^, respectively, during the first cycle (based on the total mass of active materials in both the cathode and anode). The full battery delivered a rate capacity of 46, 45, 39, and 36 mAh g^−1^ at 0.2, 0.3, 0.4, and 0.5 A g^−1^ after 3 cycles, respectively, and it recovered to 42 mAh g^−1^ when the current density was returned to 0.2 A g^−1^ (Figure 6e). Although the capacity achieved by the device is not significantly high, the Bi_2_Te_3_@PPy//LMP full battery delivers a maximum energy density of 89 Wh kg^−1^ at a power density of 355 W kg^−1^, and a maximum power density of 1805 W kg^−1^ at an energy density of 29 Wh kg^−1^, as shown in Figure 6f. The achieved performance surpass many reported low‐cost AAIBs based on W_18_O_49_//CuFe‐PBA,^[^ ^71^ ^]^ Eutectic‐treated Al//FeHCF,^[^ ^72^ ^]^ 9,10‐anthraquinone//K_2_CuFe(CN)_6_,^[^ ^73^ ^]^ TiO_2_//CuHCF,^[^ ^74^ ^]^ Al//KNHCF,^[^ ^75^ ^]^ Al//graphite,^[^ ^76^ ^]^ as well as several pioneering works on non‐aqueous batteries with carbon‐based cathodes, such as pyrolytic graphite,^[^ ^77^ ^]^ natural graphite flakes,^[^ ^78^ ^]^ and kish graphite flakes.^[^ ^79^ ^]^ Most significantly, long‐term cycling demonstrated a lifespan of over 1000 cycles at 0.5 A g^−1^ with no decay in specific capacity, maintaining a coulombic efficiency of ≈105% (Figure 6g). A coulombic efficiency >100% was achieved, which may be attributed to the partial oxidation of Bi_2_Te_3_ into BiOCl during discharge, which contributes to a discharge capacity slightly surpassing the preceding charge capacity.

The rechargeability of AAIBs is significantly limited when Al is used as an anode due to the formation of an insulating and passivating Al_2_O_3_ layer in the aqueous electrolyte, which greatly hinders Al^3+^ transport during subsequent Al stripping and plating.^[^ ^22^ ^]^ While higher potentials can drive ion transport through the alumina layer, they also trigger hydrogen evolution and corrosion reactions, continuously depleting the aqueous electrolyte and degrading the Al anode.^[^ ^20^, ^22^ ^]^ Therefore, we have successfully demonstrated aluminum‐free AAIBs employing the Bi_2_Te_3_@PPy anode and LiMnPO_4_ cathode. The distinctive topological insulating characteristics of the anode, combined with the high electrochemical performance of LiMnPO_4_ in AlCl_3_ electrolyte, contribute significantly to achieving highly reversible and stable ion storage with a long lifespan.

## 3 Conclusion

In summary, to tackle the key obstacles of hydrogen evolution and corrosion reactions at the Al anode, we have innovatively designed topological insulator Bi_2_Te_3_@PPy nanodiscs via a simple solvothermal method, demonstrating their potential as an anode in AAIBs. Leveraging the topological quantum surface states and the interconnected PPy network within Bi_2_Te_3_ nanodiscs, the Bi_2_Te_3_@PPy composite synergistically integrates structural and compositional strategies. This results in enhanced electrical conductivity and an increased number of active sites, which promote the ion migration rate and surface adsorption capacity. Benefiting from these properties, the Bi_2_Te_3_@PPy anode exhibits high specific capacity, excellent rate capability, and a distinct discharging plateau, even at high current densities. Our findings reveal that the TI, Bi_2_Te_3_, exhibits characteristics corresponding to the surface adsorption/desorption of hydrated Al^3+^ and the ion insertion/extraction of H_3_O^+^. Hence, this study opens new avenues for advancing AAIB technology and provides fresh insights into the intercalation chemistry of cations (H^+^, H_3_O^+^, Al^3+^, and hydrated Al^3+^) in aqueous electrolytes.

## Conflict of Interest

The authors declare no conflict of interest.

## Author Contributions

M.P. and P.D. conceived the project and designed the experiments. P.D. carried out the material preparation, characterization, electrochemical experiment, and data analysis. P.L. carried out the DFT study. M.P., M.O., P.D., and P.L. wrote the manuscript. M.P. supervised the entire project and provided research direction. All the authors contributed to the discussion and manuscript preparation.

## Supporting information



Supporting Information

## Data Availability

The data that support the findings of this study are available from the corresponding author upon reasonable request.
