# Supplementary material for: Topological Insulator Bi2Te3 Anode for Aqueous Aluminum‐Ion Batteries: Unveiling the Role of Hydronium Ions
Source: Adv Sci (Weinh). 2025 Jul 6;12(37):e07255. doi: 10.1002/advs.202507255 (PMC12499396; doi:10.1002/advs.202507255)
Supplement: Supplementary file 1 — Supporting Information [file ADVS-12-e07255-s001.docx]

**Supporting Information**

**Topological Insulator Bi_2_Te_3_ Anode for Aqueous Aluminum-ion Batteries: Unveiling the Role of Hydronium Ions**

Puja De^a^, Petr Lazar^b^, Michal Otyepka^b,c^, and Martin Pumera^a,d,e^*

^a^Faculty of Electrical Engineering and Computer Science, VSB - Technical University of Ostrava, 17. listopadu 2172/15, 70800 Ostrava, Czech Republic

^b^Regional Centre of Advanced Technologies and Materials, The Czech Advanced Technology and Research Institute (CATRIN), Palacký University Olomouc, Šlechtitelů 27, 779 00 Olomouc, Czech Republic

^c^IT4Innnovations, VSB-Technical University of Ostrava, 17. listopadu 2172/15, 708 00 Ostrava-Poruba, Czech Republic

^d^Future Energy and Innovation Laboratory, Central European Institute of Technology, Brno University of Technology, Purkyňova 123, 61200 Brno, Czech Republic

^e^Department of Medical Research, China Medical University Hospital, China Medical University, No. 91 Hsueh-Shih Road, Taichung 40402, Taiwan

* Author for correspondence: M. Pumera, martin.pumera@vsb.cz

Experimental Section

*Chemicals:* Potassium hydroxide (KOH, ≥85%), bismuth(III) nitrate pentahydrate (Bi(NO_3_)_3_·5H_2_O, ≥99.99%), Tellurium(IV)oxide (TeO_2_, ≥99.99%), ethylene glycol (EG, HOCH_2_CH_2_OH, 99%), activated carbon, Polyvinylidene fluoride ((CH_2_CF_2_)n), N-Methyl-2-pyrrolidone (C_5_H_9_NO, ≥99.0%), polyvinyl pyrrolidone (PVP), Methyl Orange (MO, C_14_H_14_N_3_NaO_3_S, 85%), pyrrole (C_4_H_5_N, 98%), Iron(III) chloride (FeCl_3_, 97%), aluminum chloride (AlCl_3_, 99%), potassium chloride (KCl, ≥99.0%), sodium chloride (NaCl, ≥99.0%), magnesium chloride (MgCl_2_, ≥98.0%), calcium chloride (CaCl_2_, ≥96.0%), and bulk Bi_2_Te_3_ were purchased from Sigma. Ethanol was of analytical grade and obtained from P-LAB, Czech Republic. All chemicals were used as received, without further purification.

*Synthesis of Nanostructures of Bi_2_Te_3_:* To prepare nanodisk of Bi_2_Te_3_, 0.4 g of PVP was first added in 18 ml of EG and stirred until completely dissolved. Afterward, 2 mL of 5 mol/L NaOH solution, 30 mmol of TeO_2_ and 20 mmol of Bi(NO_3_)_3_·5H_2_O were dissolved into the above solution. After stirring for 60 minutes, the mixed solution was transferred into a 50 mL Teflon-lined stainless-steel autoclave and then heated at 180 °C for 6 hours. Finally, the obtained Bi_2_Te_3_ nanodisks suspension was washed with ethanol and dried in an oven at 60 °C.

**For the synthesis of Bi_2_Te_3_ nanoparticles,** the same procedure was followed, except ethanol was used as the reaction medium instead of EG.

*Synthesis of poypyrrole (PPy):* Polypyrrole was synthesized using the chemical oxidation method of the monomer. To begin, 12 mg of methylene orange (MO) was mixed with 20 mL of deionized (DI) water in a beaker and stirred to obtain a homogeneous solution. MO acted as the surfactant in this process. While continuously stirring in an ice bath, 60 g of the chemical oxidant FeCl₃ was added to the MO solution, followed by the addition of 26 µL of pyrrole. The entire mixture was left under continuous stirring in the ice bath for 24 hours. Afterward, the solution was filtered and washed several times with DI water and ethanol. Finally, the precipitate was dried overnight at 60 °C to achieve the desired morphology of PPy.^1^

*Synthesis of Bi_2_Te_3_@PPy* nanodisk*:* For the synthesis of Bi_2_Te_3_@PPy nanodisks, the MO, FeCl_3_, and pyrrole solution was first prepared as discussed in the synthesis of polypyrrole. Another solution was then prepared by dissolving a specific amount (70-80 mg) of Bi_2_Te_3_ nanodisks in 10 mL of DI water, and this solution was added dropwise to the previously prepared solution. The entire mixture was left under continuous stirring in an ice bath for 24 hours. Afterward, the obtained Bi_2_Te_3_@PPy nanodisks suspension was washed with ethanol several times and dried overnight in an oven at 60 °C.

*Materials Characterizations:* **The synthesized materials were morphologically characterized by Scanning Electron Microscope (SEM, FEI VERIOS 460L)**. The phase confirmation of the as-prepared products was obtained using a powder X-ray diffractometer Rigaku D/tex Ultra 250 with Cu K_α_ radiation (λ = 0.15406 nm). The X-ray photoelectron spectroscopy (XPS) measurements were performed using the Nexsa G2 XPS system (Thermo Fisher Scientific) with a monochromatic source (Al-Kα) and a photon energy of 1486.7 eV. All the spectra were measured in the vacuum of 2 × 10^−7^ Pa and at a temperature of 20 °C. The specific surface area of the products was determined using the Brunauer-Emmett-Teller (BET) method (adsorption set-up 3Flex, Micromeritics). FT-IR spectra were measured using a Thermo Scientific Nicolet iS10 FT-IR spectrometer.

*Electrochemical Measurements:* The electrochemical properties of the prepared materials were evaluated using both three-electrode and two-electrode configurations. Nanoparticles and nanodisks of Bi_2_Te_3_, bulk Bi_2_Te_3_, and Bi_2_Te_3_@PPy were mixed with activated carbon and polyvinylidene fluoride (PVDF) in N-methyl-2-pyrrolidone (NMP) at a mass ratio of 8:1:1. The resulting slurry was then coated onto a graphite sheet and dried overnight in a vacuum oven at 60 °C to prepare the working electrode. The mass loading of each prepared electrode was maintained at ~1 mg cm^-2^ during the galvanostatic charge-discharge measurements. A 0.5 M aqueous solution of AlCl_3_ was used as the electrolyte. In the three-electrode configuration, platinum wire and a saturated Ag/AgCl electrode were used as the counter and reference electrodes, respectively. In the two-electrode configuration, Bi_2_Te_3_@PPy-coated graphite sheet served as the negative electrode, while a LiMnPO_4_-coated graphite sheet acted as the positive electrode. Cyclic voltammetry, galvanostatic charge-discharge (GCD), and electrochemical impedance spectroscopy (EIS) measurements were performed using an Metrohm Autolab electrochemical workstation employing NOVA 2.1.6 software in a 20 mL cell at room temperature with Ag/AgCl (1 M KCl) and Pt as a reference and counter electrodes, respectively.

*Density Functional Theory (DFT) Calculations:* DFT calculations were performed using the projector-augmented wave method implemented in the Vienna Ab initio Simulation Package (VASP).^2-3^ The energy cutoff for the plane-wave expansion was set to 500 eV. We used optimized van der Waals functional optB86b-vdW functional,^4^ which provides balanced description of van der Waals as well as covalent and ionic bonding. We first optimized the crystal structure of Bi_2_Te_3_; we obtained the lattice parameters of *a*=4.36 Å and *c*=30.31 Å, in a good agreement with experimental values. The diffusion was modelled using a 2×2×1 supercell in connection with 6×6×1 k-point grid. First, the equilibrium position of a specie (H^+^, H_3_O^+^, Al^3+^, Al(H_2_O)_4_^3+^) within the van der Waals gap of Bi_2_Te_3_ was found by atomic relaxation until the forces were below 0.01 eV/Å. For each ion, relaxation was performed for several starting positions within vdW gap of Bi_2_Te_3_. Then, the diffusion barrier between two neighbouring equilibrium positions was calculated using the nudged elastic band method. The supercell was allowed to shrink/expand along the z direction (i.e., to change the *c* lattice parameter) during both relaxation and barrier calculation.

The adsorption energy $E_{ad}$was calculated as:

|  | $E_{ad}=E_{Bi2Te3+X}-E_{Bi2Te3}-E_{X}$*.* | (1) |
| --- | --- | --- |

Here, $E_{Bi2Te3}$ corresponds to the total energy of the Bi_2_Te_3_ surface represented by quintuplet layer slab, $E_{Bi2Te3+X}$ is the total energy of given specie (H_3_O^+^, Al(H_2_O)_4_^3+^) adsorbed on the Bi_2_Te_3_ slab and $E_{X}$the total energy of respective isolated species. The total energies $E_{Bi2Te3+X}$ and $E_{X}$ were calculated for neutral systems to avoid the issues because of the periodic replica of the added charge.

For DFT calculations, we considered [Al(H_2_O)_4_]^3+^ instead of [Al(H_2_O)_6_]^3+^ because its planar arrangement fit better into van der Waals gap between layers of Bi_2_Te_3_. The assumption of a planar [Al(H_2_O)_4_]^3+^ geometry was based on steric considerations, as the fully hydrated [Al(H_2_O)_6_]^3+^ ion is too large to fit within the van der Waals (vdW) gaps of Bi_2_Te_3_. Figure S1 showing the [Al(H_2_O)_6_]^3+^ ion inserted into a vdW gap of Bi_2_Te_3_, clearly indicating that its presence would likely lead to structural delamination.


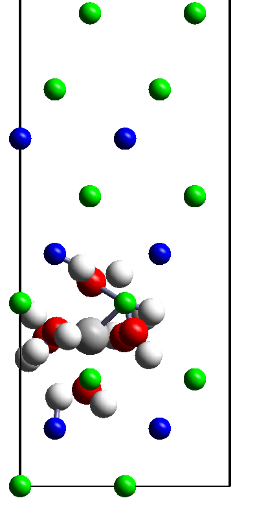


*Figure S1: The geometry of [Al(H_2_O)_6_]^3+^ ion inserted into a vdW gap of Bi_2_Te_3_.*

In contrast, the partially hydrated [Al(H_2_O)_4_]^3+^ ion with a planar geometry fits more plausibly into the layered structure of Bi_2_Te_3_. The flexibility of the water ligands also allows for partial accommodation and diffusion of this ion within the vdW gaps.

We additionally carried out DFT calculations to assess the electrostatic polarization induced by the intercalated [Al(H_2_O)_4_]^3+^. The polarization charge was computed as the difference between the charge density of the fully intercalated system and the sum of charge densities of the isolated Bi_2_Te_3_ host and the hydrated Al^3+^ ion (Figure S2). These results reveal significant polarization localized around the [Al(H_2_O)_4_]^3+^ species, supporting its electrostatically driven interaction with the host lattice.

*Figure S2: The polarization charge of intercalated [Al(H_2_O)_4_]^3+^ computed as the difference between the charge density of the fully intercalated system and the sum of charge densities of the isolated Bi_2_Te_3_ host and the hydrated Al³⁺ ion.*


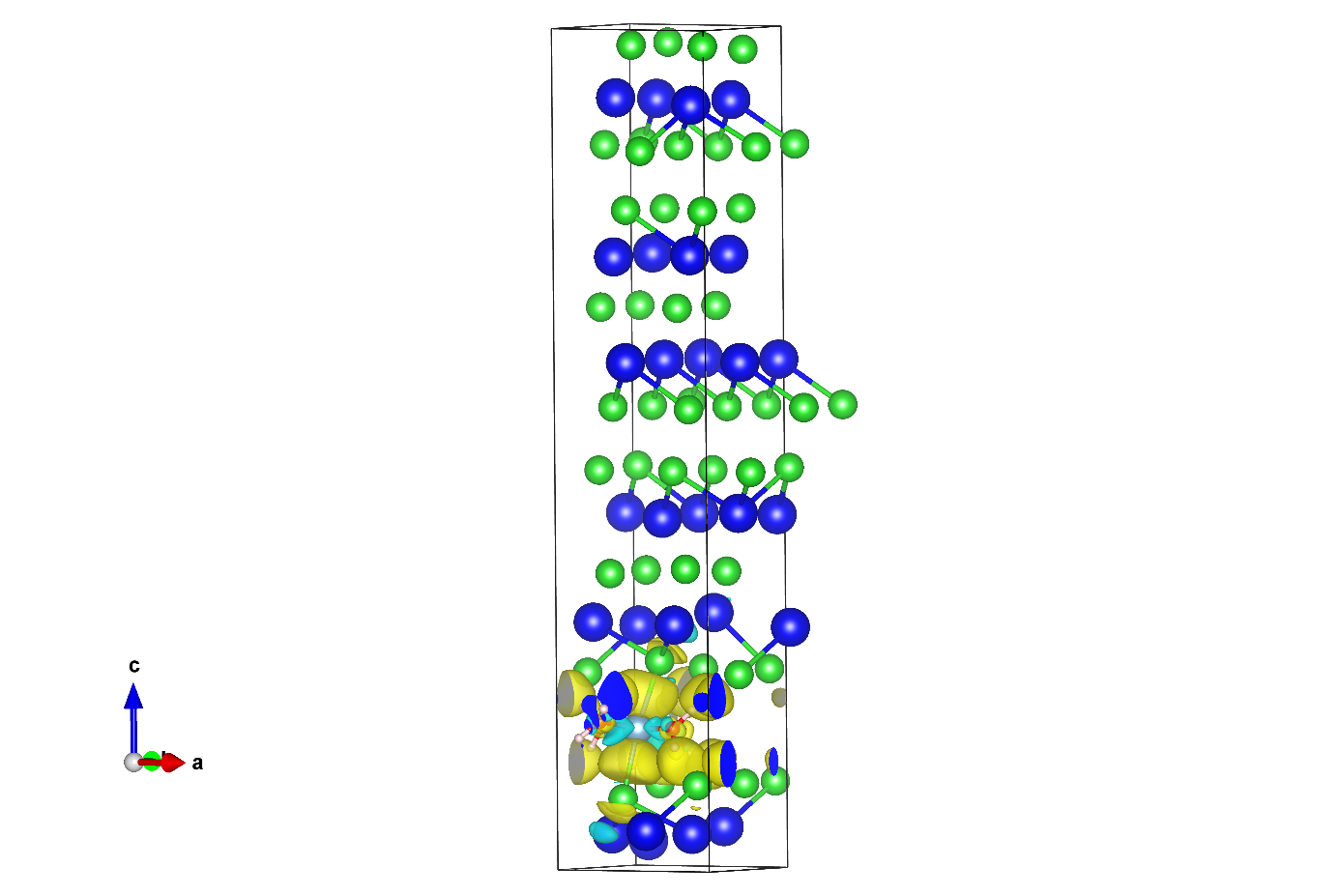


To provide an estimate of the energy cost associated with partial desolvation, we compared the total energies of the isolated [Al(H_2_O)_4_]^3+^ and [Al(H_2_O)_6_]^3+^ species in vacuum. The energy difference for the transformation ${[Al{(H_{2}O)}_{6}]}^{3+}\to{[Al{(H_{2}O)}_{4}]}^{3+}+2H_{2}O$ amounts to 141.5 kcal/mol.

*Figure S3: Scanning electron microscopy image of the synthesized (a) Bi_2_Te_3_ nanodisks, (b-c) Bi_2_Te_3_ nanoparticles at different magnifications, and (b) commercial bulk Bi_2_Te_3_.*


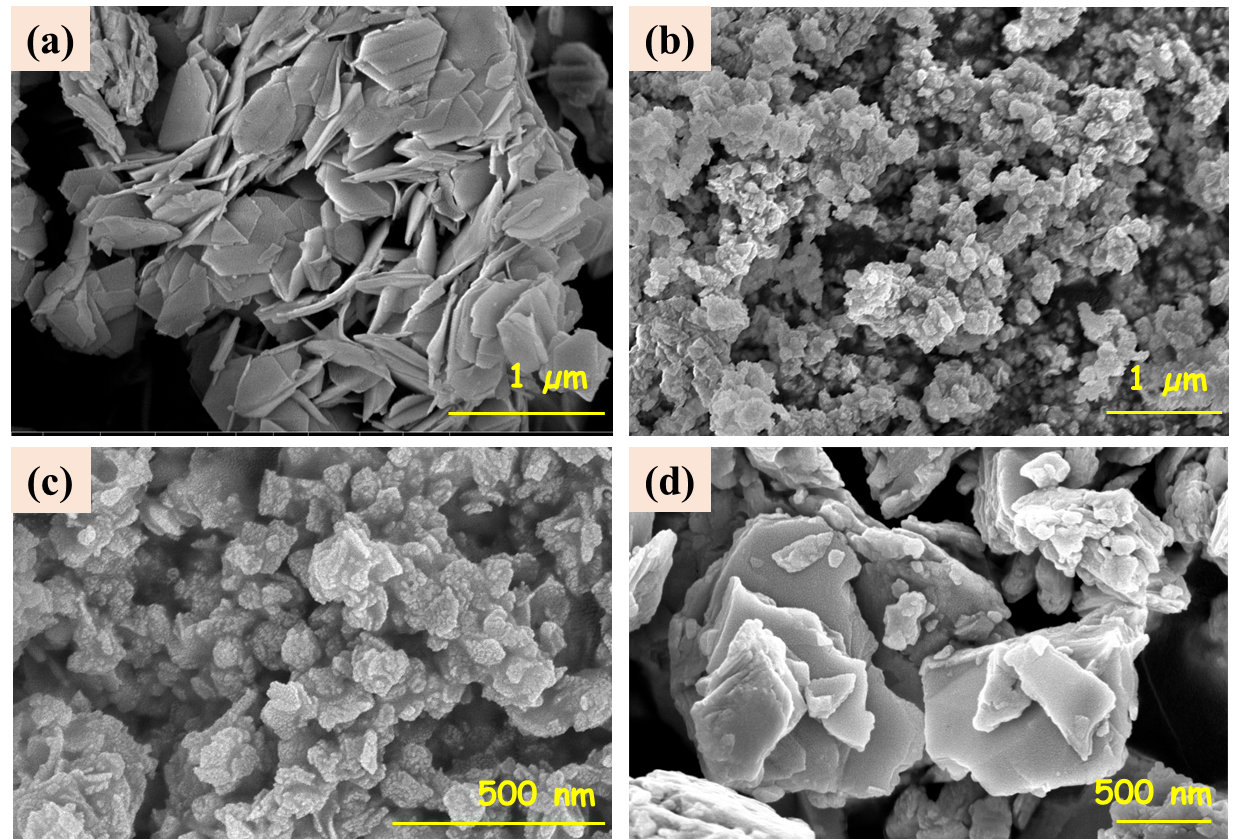


*Figure S4: Scanning electron microscopy image of the synthesized (a) polypyrrole (PPy), and (b) Bi_2_Te_3_@PPy nanodiscs.*


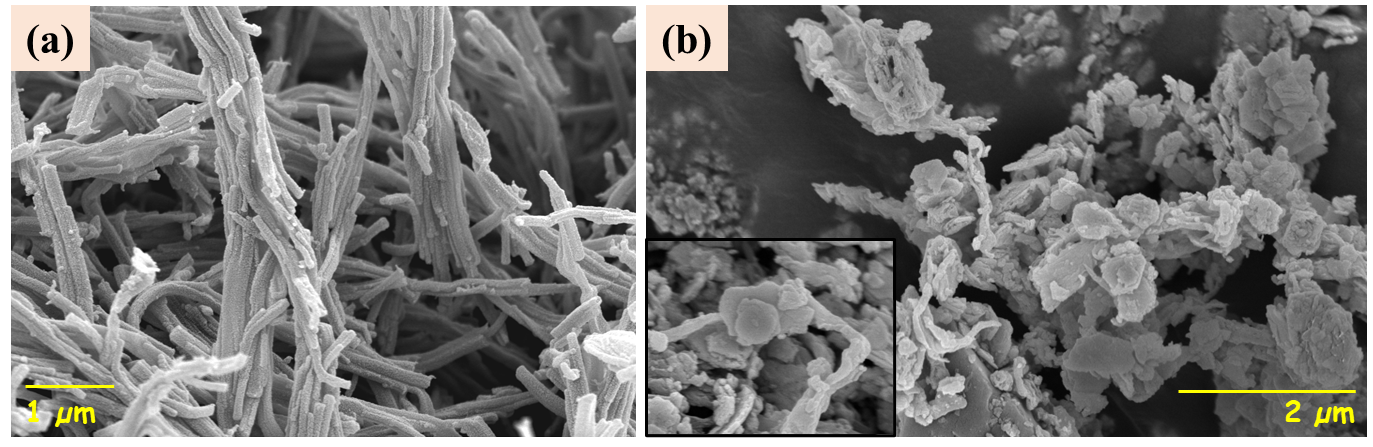

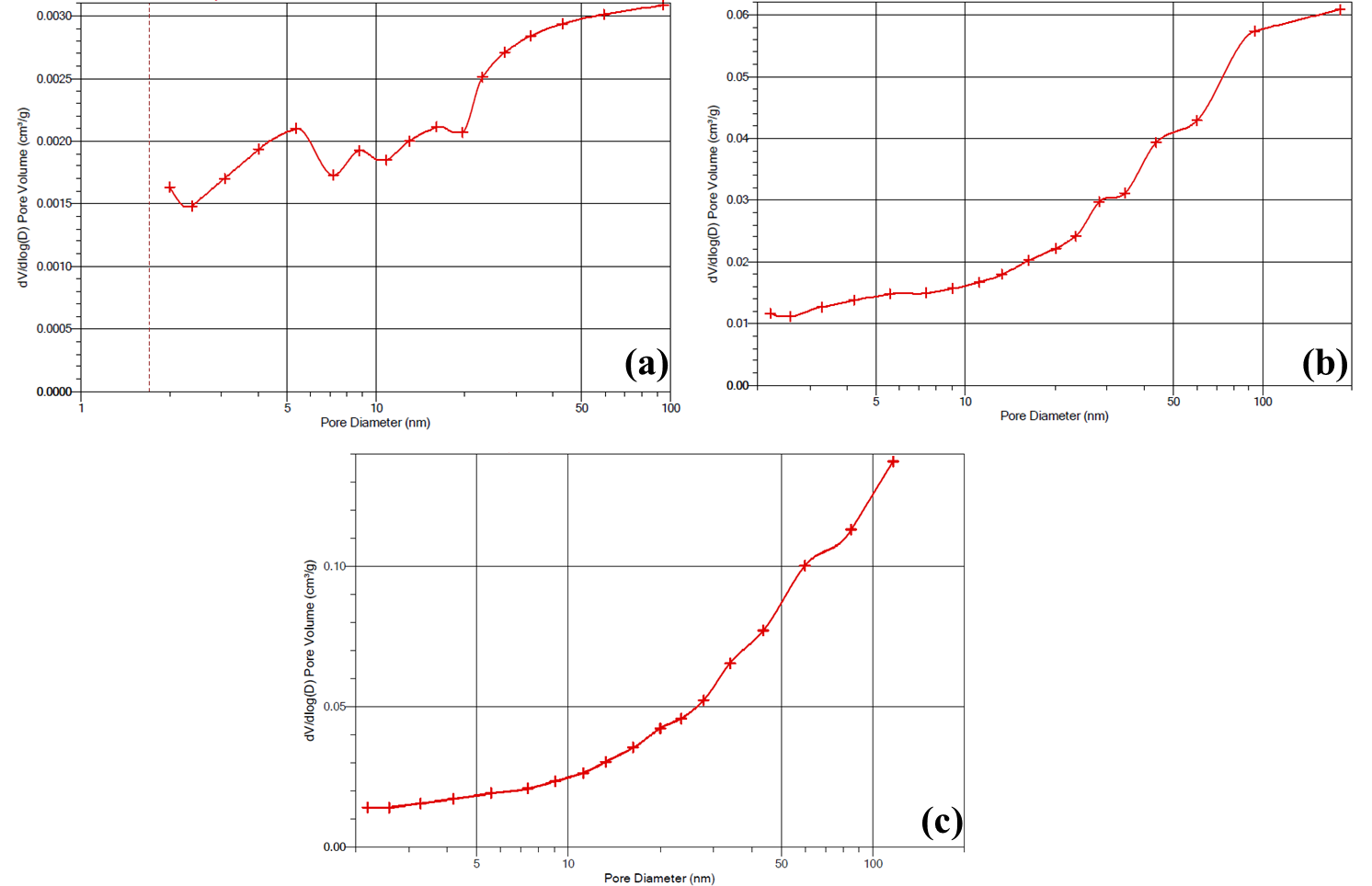


*Figure S5: BJH adsorption dV/dlog(D) pore volume of the (a) bulk Bi_2_Te_3_ and the synthesized (b) Bi_2_Te_3_ nanoparticles and (c) nanodisks.*

*Table S1: Specific surface area and pore volume of the bulk and the synthesized Bi_2_Te_3_ nanomaterials.*

| Material | Specific surface area (m^2^ g^-1^) | Pore volume (mm^3^ g^-1^) |
| --- | --- | --- |
| Bulk | 2 | 5 |
| Nanoparticle | 13 | 71 |
| Nanodisk | 25 | 141 |

*Figure S6: XPS survey spectra of Bi_2_Te_3_ nanodisks.*

The CV profiles for the different morphologies at scan rates ranging from 1 to 5 mV s^-1^ are shown in Figure S7. Generally, with increasing scan rate, the oxidation and reduction peak intensities are expected to increase according to the equation $i=av^{b}$.^5^ However, in the present case, we observed that for the nanoparticle and nanodisk materials, the peak current responses for both the cathodic and anodic redox peaks do not follow this relationship. With increasing scan rate, the peak intensity remained either constant or slightly decreased.^6-7^ In contrast, for the bulk sample, the peak intensity increased with increasing scan rate. This suggests that in the nanostructured samples of Bi_2_Te_3_, the higher surface-to-volume ratio promotes surface conversion reactions due to greater accumulation of Cl⁻ ions at higher scan rates, leading to the formation of BiOCl in the aqueous environment. Although surface conversion reactions occur, the high surface-to-volume ratio of the nanostructured Bi_2_Te_3_ provides more topological surface states, which are inherently active in charge transport and surface adsorption.^8-10^ These properties of the nanostructured Bi_2_Te_3_ promote the facile intercalation/deintercalation of ions from the electrolyte, resulting in much more intense oxidation/reduction peaks in the nanostructures Bi_2_Te_3_ compared to the bulk Bi_2_Te_3_.

*Figure S7: Cyclic voltammetry profile of (a) bulk Bi_2_Te_3_, (b) Bi_2_Te_3_ nanoparticles, and (c) Bi_2_Te_3_ nanodisks at various scan rates in 0.5 M AlCl_3_ electrolyte.*


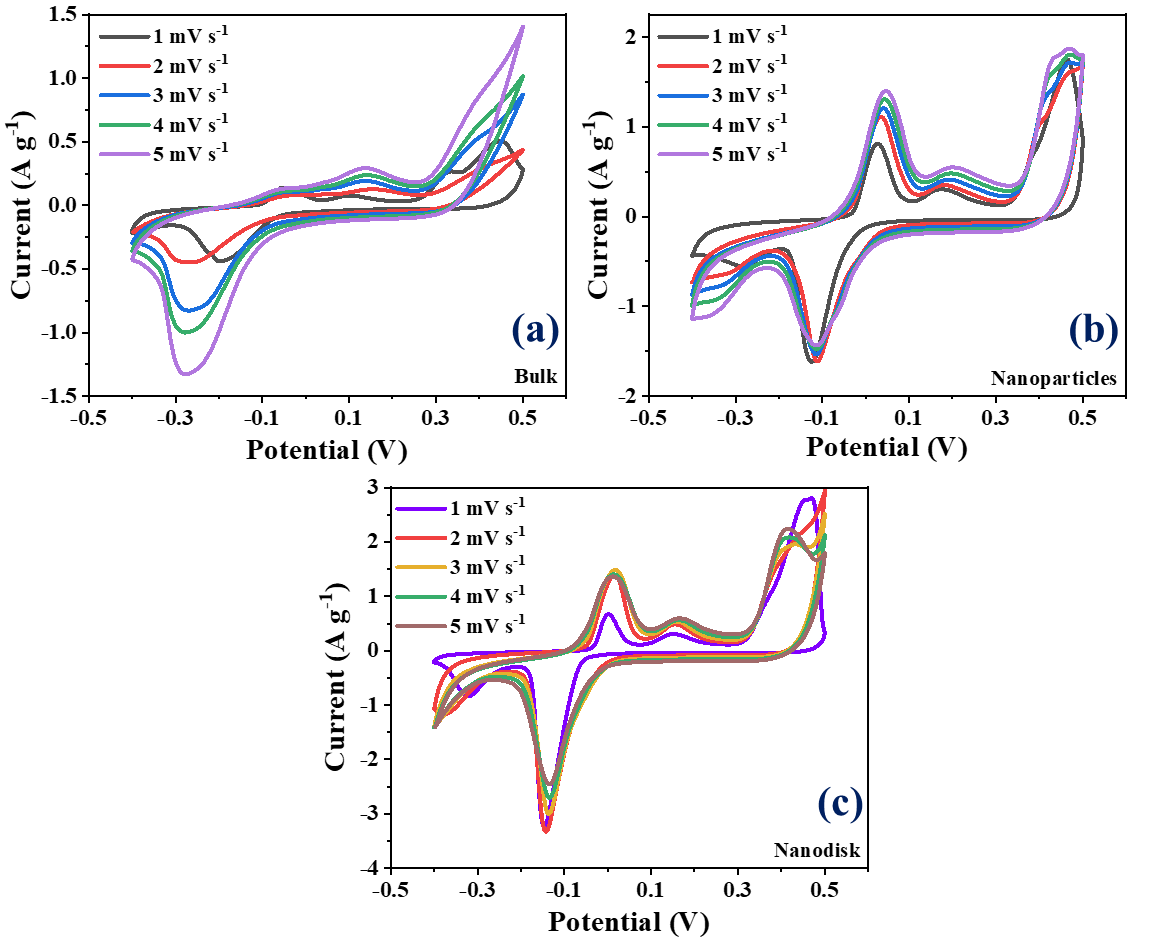


*Figure S8: Nyquist plot of Bi_2_Te_3_ nanodisk in 0.5 M AlCl_3_ solution (in ethylene glycol organic solvent).*

*Electrochemical performance in aprotic solvents:*

Ethylene glycol (EG) is a protic solvent. Hence, we can’t entirely rule out the proton involvement in EG-AlCl_3_ electrolyte. However, we selected EG because it is a suitable electrolyte solvent and inhibits proton ionization.^11^ EG has been successfully employed in various studies,^11-12^ where it demonstrated excellent compatibility of EG with metal-ion electrolytes. Notably, the concentration of free protons in the EG-AlCl_3_ system is expected to be significantly lower than in the water-AlCl_3_ system. When aluminum chloride (AlCl_3_) dissolves in water, the Al^3+^ ions become hydrated, forming the hexaaqua complex [Al(H_2_O)_6_]^3+^. Due to the high charge density of the aluminum ion, the water molecules in the complex become polarized. This can lead to hydrolysis, where a proton (H^+^) is released from the water molecules attached to Al^3+^, creating hydronium ions (H_3_O^+^) in the solution. In contrast, EG molecules coordinate strongly with Al^3+^ through their hydroxyl groups, forming stable solvated complexes that suppress the hydrolysis of Al^3+^ and minimize the release of free protons. This results in a lower proton concentration in EG-AlCl_3_ solutions, thereby reducing proton-coupled redox activity.

*Figure S9: (a) AlCl_3_ solution in DMF, EG, and NMP solvents; (b) AlCl_3_ solution in DMSO and acetonitrile solvents; CV profile of Bi_2_Te_3_ nanodisk at a scan rate of 1 mV s^-1^ in (c) AlCl_3_-NMP (d) AlCl_3_-DMF, and (e) AlCl_3_-EG.*


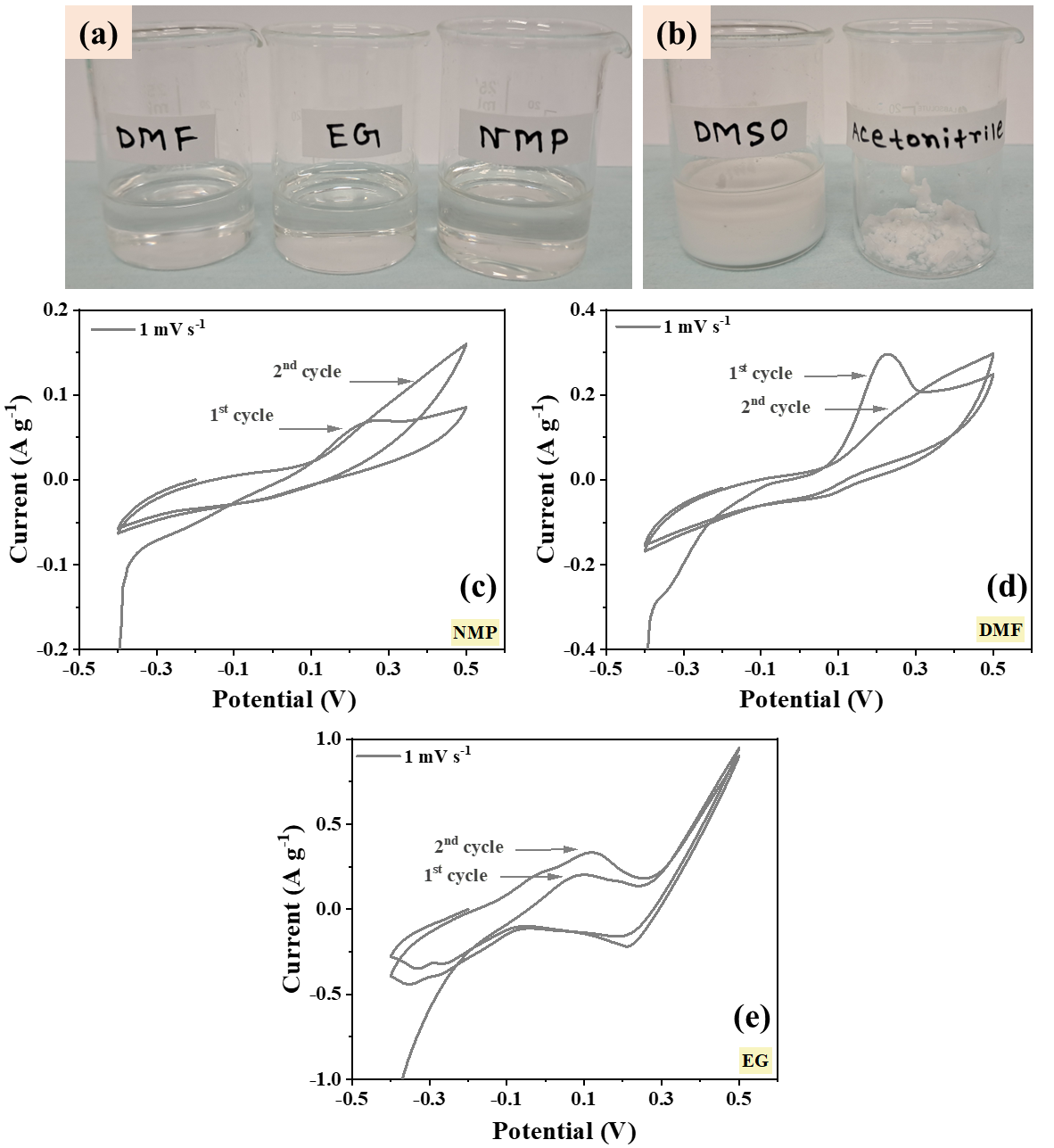


While aprotic solvents such as dimethyl sulfoxide (DMSO), N, N-dimethylformamide (DMF), N-methylpyrrolidone (NMP), and acetonitrile could theoretically minimize proton involvement, their use as practical battery electrolytes remains relatively unexplored and challenging due to issues like poor salt solubility, low ionic conductivity, and unstable solid-electrolyte interphase.^13-15^ In contrast, EG offers a stable electrochemical window and efficient solvation capability for multivalent cations, making it suitable for evaluating the redox behavior in our system. We also attempted to evaluate the electrochemical performance of Bi_2_Te_3_ nanodisks in DMSO, DMF, NMP and acetonitrile. However, upon adding AlCl_3_ salt to DMSO and acetonitrile, precipitation and phase separation occurred, likely due to poor solubility of AlCl_3_ in these solvents, as shown in Figure S9b. The CV profiles obtained in NMP and DMF are presented in Figure S9c-d, showing a prominent oxidation peak appearing at a potential closely matching that observed in the EG-AlCl_3_ electrolyte. However, this redox behavior is unstable with very low current response confirming that these aprotic solvents are not suitable for reliable electrochemical evaluation.

*Figure S10. Nyquist plot of bulk and nanostructured Bi_2_Te_3_.*

*Table S2: Equivalent series resistance values (ESR) of Bi_2_Te_3_ electrodes.*

| *Material* | *Equivalent series resistance (in Ω)* |
| --- | --- |
| Bulk Bi_2_Te_3_ | ~ 3.5 |
| Bi_2_Te_3_ nanoparticles | ~ 2.6 |
| Bi_2_Te_3_ nanodisk | ~ 1.1 |

*Figure S11. Electrochemical study of Bi_2_Te_3_ nanodisk in various electrolytes. CV profiles of Bi_2_Te_3_ nanodisks at 1 mV s^-1^ in 0.5 M (a) NaCl, (b) KCl, (c) CaCl_2_, and (d) MgCl_2_, electrolytes; Galvanostatic charge-discharge profile of Bi_2_Te_3_ nanodisks in 0.5 M (e) NaCl, (f) KCl, (g) CaCl_2_, and (h) MgCl_2_ electrolyte at 0.5 A g^-1^; (i) comparison of achieved specific capacity at 0.5 A g^-1^.*


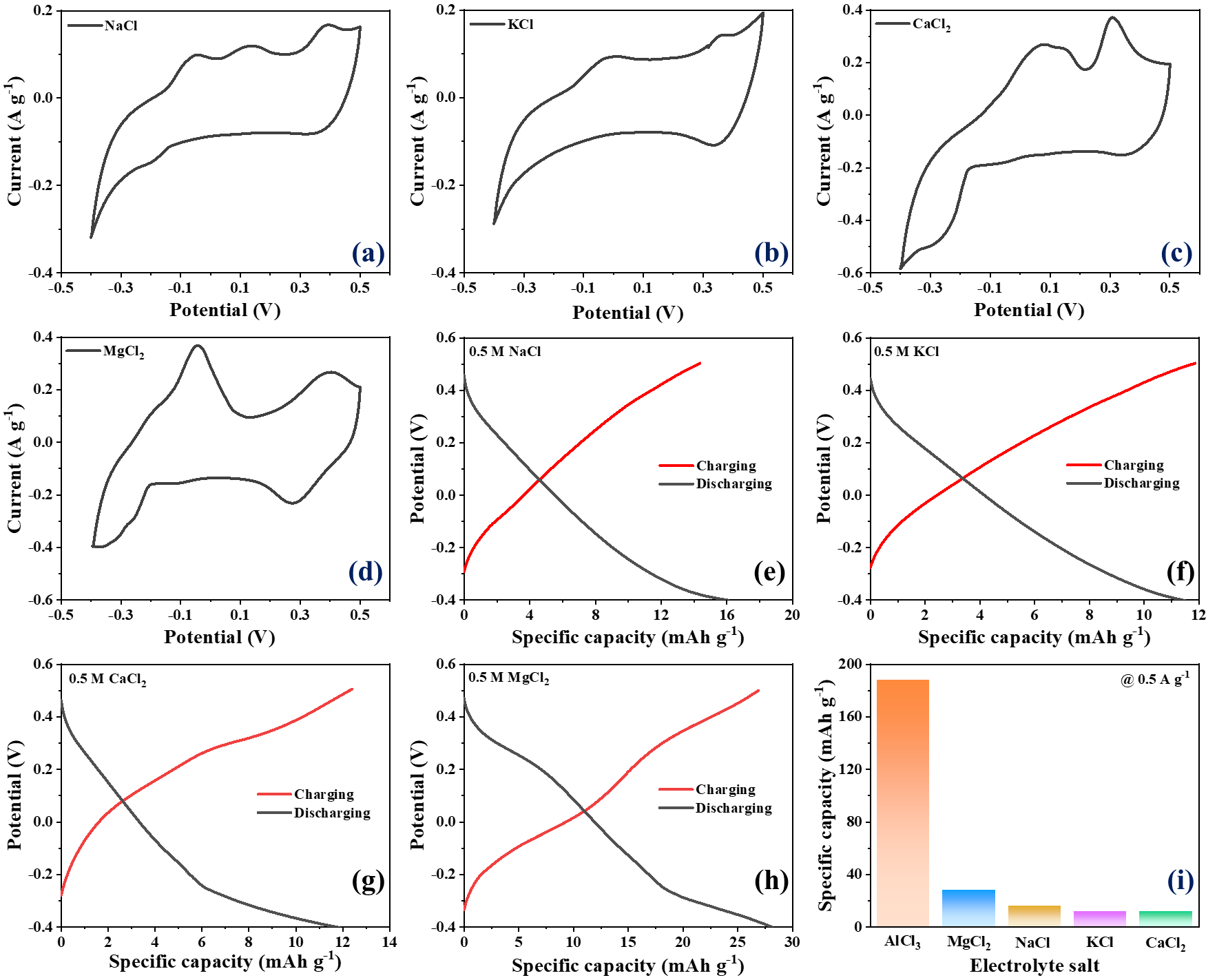


*Table S3: Measured pH values at room temperature (21.4 °C) and calculated proton concentrations of various electrolyte solutions.*

| Electrolyte | pH value | Proton concentration (mol/L) |
| --- | --- | --- |
| 0.5 M AlCl_3_ | 3.903 | $1.25\times{10}^{-4}$ |
| 0.5 M MgCl_2_ | 8.866 | $1.36\times{10}^{-9}$ |
| 0.5 M NaCl | 6.252 | $5.59\times{10}^{-7}$ |
| 0.5 M KCl | 6.059 | $8.72\times{10}^{-7}$ |
| 0.5 M CaCl_2_ | 6.030 | $9.33\times{10}^{-7}$ |
| 0.5 M HCl | 1.217 | $6.06\times{10}^{-2}$ |
| 0.5 M H_2_SO_4_ | 1.171 | $6.74\times{10}^{-2}$ |

*Change of pH in 0.5 M AlCl_3_ electrolyte during charge-discharge:*

The initial pH of the AlCl_3_ aqueous solution was 3.903. After the first charging, the pH increased to 4.199, consistent with the proton intercalation into the electrode material, which reduces the free proton concentration in the electrolyte. During the subsequent discharge, the pH slightly decreased to 4.183, as protons were deintercalated from the electrode into the electrolyte solution. These observations suggest that pH fluctuations occur during the charge-discharge. It is important to note that no water splitting was observed at the counter electrode during the electrochemical measurements, which could otherwise lead to additional pH changes in the electrolyte.

Though, protons are involved in the storage mechanism, the variation of pH during charge-discharge is not highly significant. This moderate pH change can be explained by calculating the total number of protons present in the electrolyte solution. The following is the number of protons present in the electrolyte solution:

Volume of the electrolyte = 10 mL

pH = 3.903

$$pH= -\log\left[ H^{+} \right]$$

$$Hence, \left[ H^{+} \right]= {10}^{-pH}={10}^{-3.903}=1.25\times{10}^{-4} mol/L$$

$$Total moles of H^{+}=\left[ H^{+} \right]\times volume=1.25\times{10}^{-4}\times0.01 mol=1.25\times{10}^{-6} mol$$

$$Number of H^{+} ions=1.25\times{10}^{-6}\times6.022 \times{10}^{23}=7.5275 \times{10}^{17}$$

Hence, a large proton reservoir is present in the prepared electrolyte solution. The pH increased to 4.199 after the first charging step, corresponding to $\sim3.806 \times{10}^{17}$protons. Hence, even a moderate change in pH results in a significant change in the total number of protons in the system. Consequently, although proton involvement in the electrochemical reaction is substantial, the overall change in electrolyte pH remains moderate.

This is also important to point out that the AlCl_3_ electrolyte acts as a buffered system due to the hydrolysis equilibrium of Al^3+^ species (${[Al{(H_{2}O)}_{6}]}^{3+}\to{[Al{(H_{2}O)}_{5}(OH)]}^{2+}+H^{+}$), which may also resist large pH variations of the electrolyte solution.^16^

*Figure S12: Cyclic-voltammetry profile of Bi_2_Te_3_ nanodisk in 0.5 M (a) H_2_SO_4_, and (b) HCl electrolyte at 1 mV s^-1^; Galvanostatic charge-discharge profile of Bi_2_Te_3_ nanodisks in 0.5 M (c) H_2_SO_4_, and (d) HCl electrolyte at 0.5 A g^-1^.*


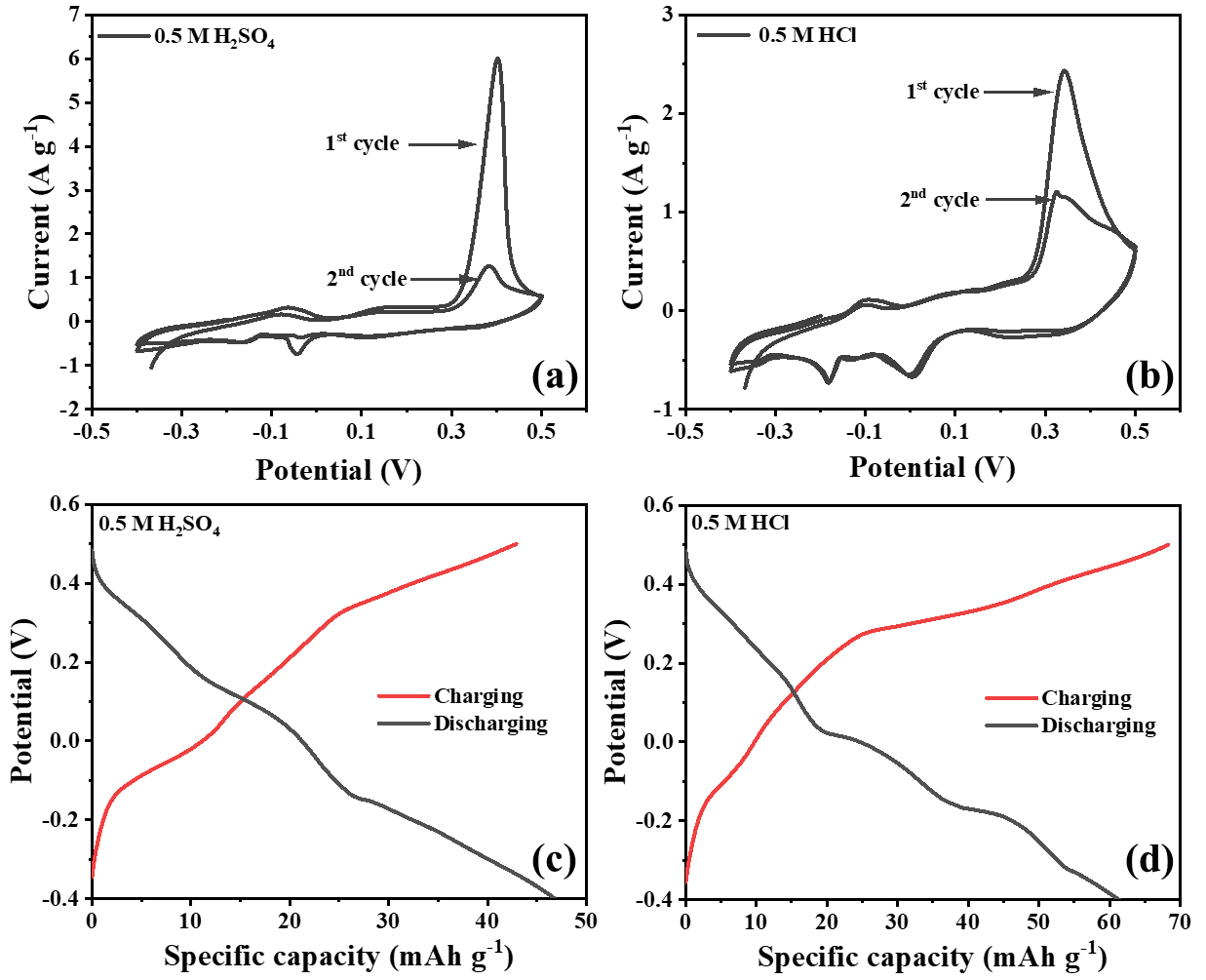

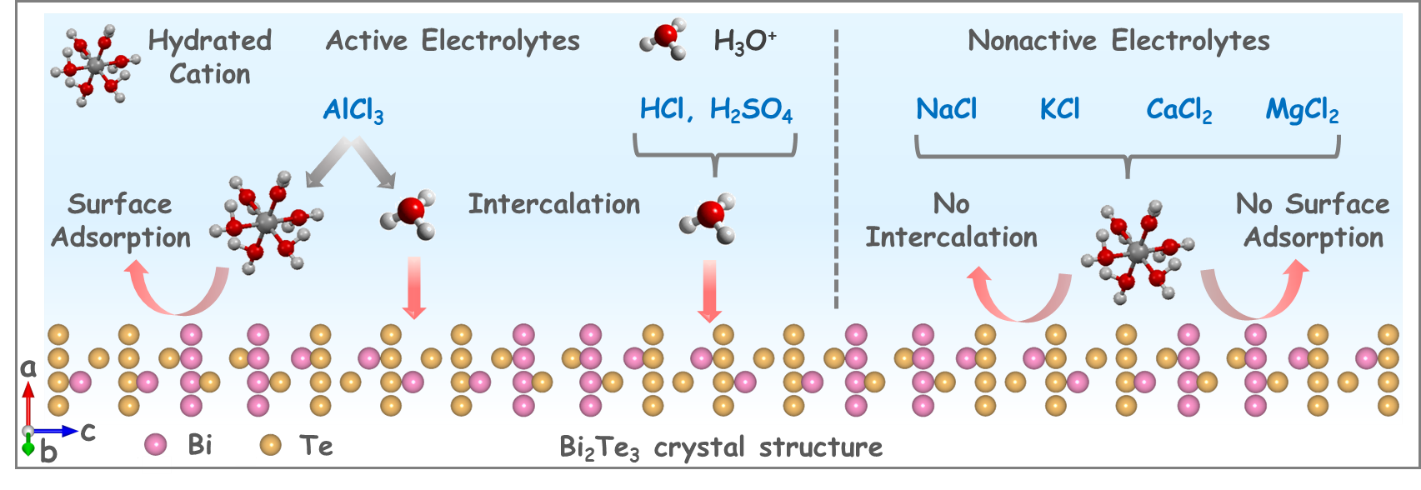


*Figure S13: Role of different cations in various electrolytes to store charges in Bi_2_Te_3_ nanodisks.*

*Figure S14: (a) CV profile at 1 mV s^-1^ and (b) GCD profile at 0.5 A g^-1^ for the nanodisk electrode of Bi_2_Te_3_ in 0.003 M HCl electrolyte.*


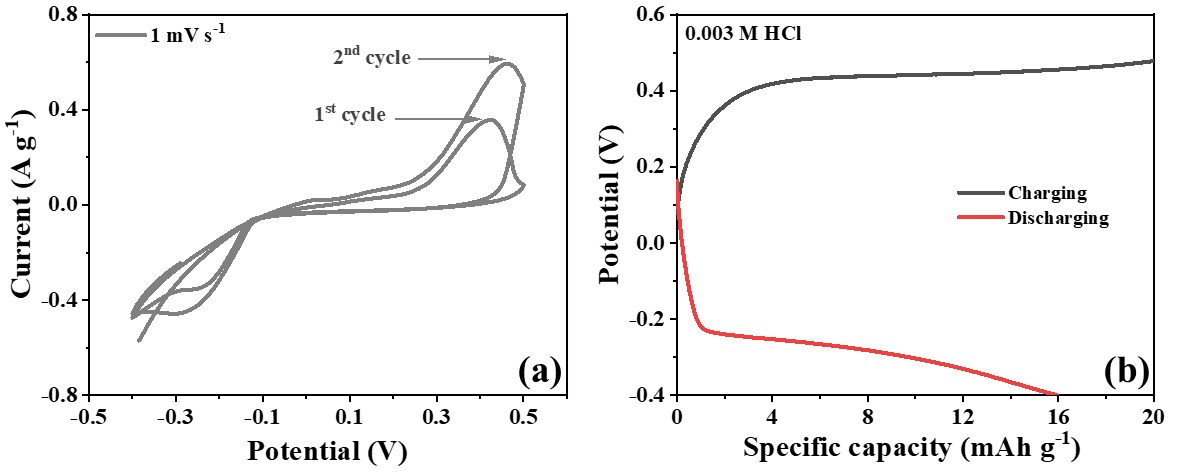


*Figure S15: Zoomed ex-situ XRD profile of Bi_2_Te_3_ nanodisk electrode at different charging-discharging states: for (a) (006) plane, and (b) (101) plane.*


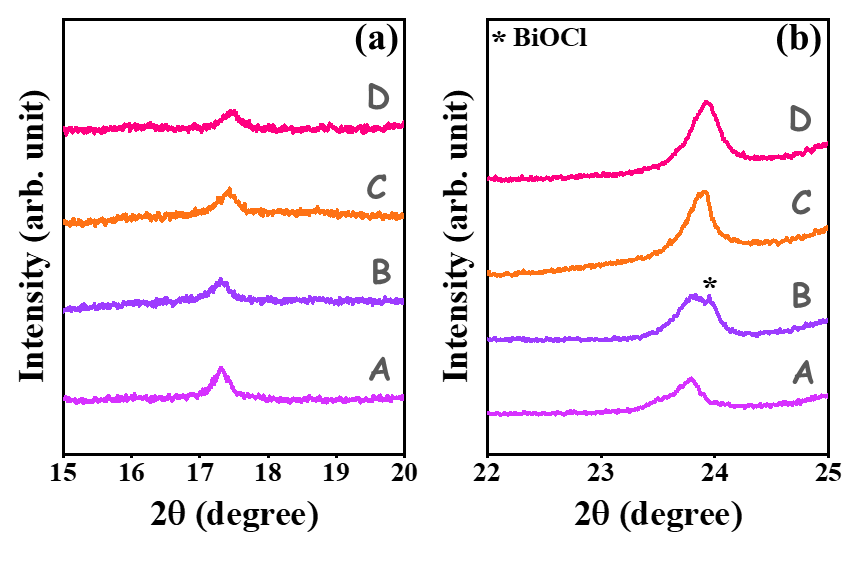

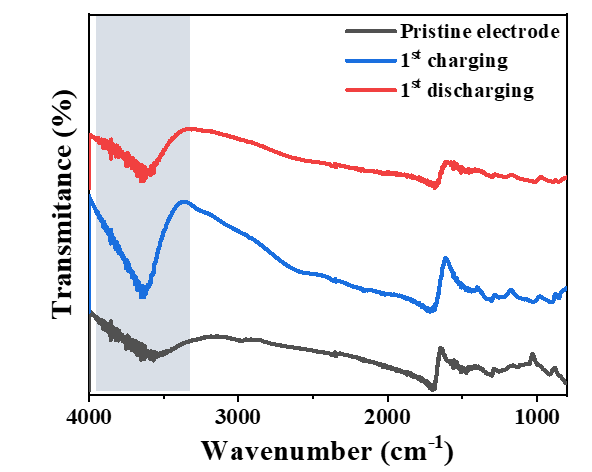


*Figure S16: Ex-situ FTIR spectra of Bi_2_Te_3_ nanodisk electrode.*


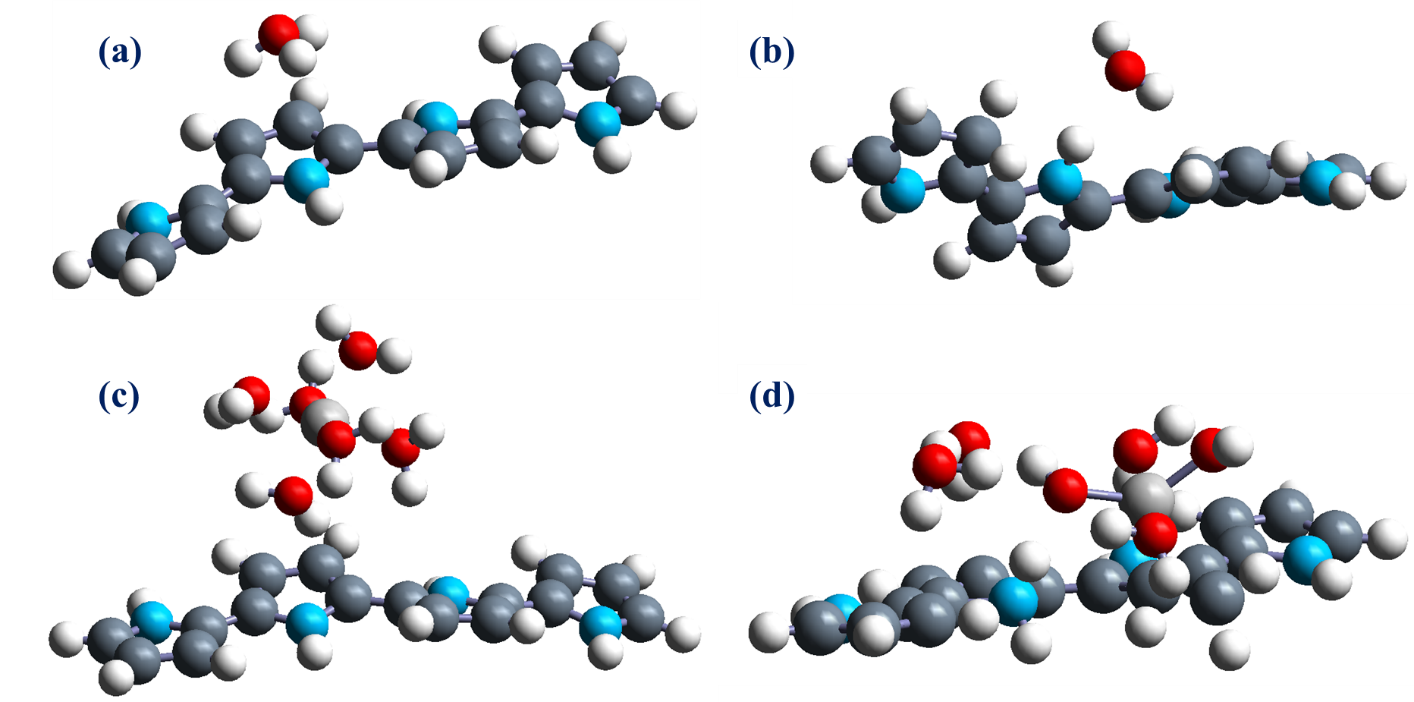


*Figure S17. Interaction of PPy with H_3_O^+^ and [Al(H_2_O)_6_]^3+^. The calculated structures of a) H_3_O^+^ adsorbed noncovalently on PPy, b) H_3_O^+^ after proton transfer to PPy, c) [Al(H_2_O)_6_]^3+^ adsorbed on PPy, and d) [Al(H_2_O)_6_]^3+^ after proton transfer to PPy* and release of water molecules*.*

*Figure S18: (a) cyclic-voltammetry profile at 1 mV s^-1^, and (b) galvanostatic charge-discharge profile at 1 A g^-1^ for pristine PPy electrode in 0.5 M AlCl_3_ electrolyte.*


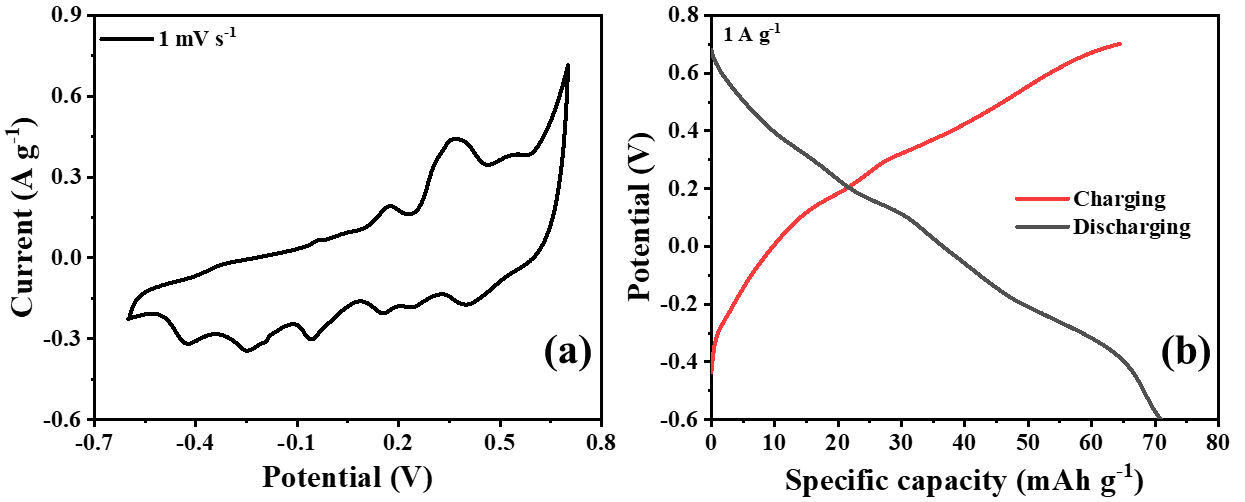


*Table S4: Comparison of performance of Bi_2_Te_3_@PPy with the existing literature.*

| Electrode material | Maximum capacity (mAh g^-1^) | Reference |
| --- | --- | --- |
| V_2_O_5_ | 140 at 0.5 A/g | 17 |
| Mn_3_O_4_ | 271 at 0.5 A/g | 18 |
| Fe-Co PBA/rGO | 112.5 at 0.5 A/g | 19 |
| ZIF 67 | 288 at 0.2 A/g | 20 |
| Bi_2_WO_6_ | 257 at 0.5 A/g | 21 |
| Bi_2_MoO_6_ | 330 at 0.5 A/g | 21 |
| oxygen-deficient α-MoO_3_ | 225 at 1 A/g | 22 |
| LiMnPO_4_ | 146 at 1 A/g | 23 |
| TiO_2_ | 249 at 3 A/g | 24 |
| LiMn_2_O_4_ | 65 at 0.8 A/g | 25 |
| MnO_2_ | 109 at 0.02 A/g | 26 |
| TiO_2_@Graphene | 50 at 6.25 A/g | 27 |
| FeVO_4_ | 350 at 0.06 A/g | 28 |
| WO_3_ | ~ 220 at 2.5 A/g | 29 |
| CuHCF | 46.9 at 0.4 A/g | 30 |
| Bi_2_Te_3_@PPy | 438 at 0.5 A/g | This work |

Synthesis of LiMnPO_4_

The synthesis of LiMnPO_4_ was carried out using protocols similar to that reported previously.^31^ A solvent mixture of ethylene glycol (EG) and deionized water (DI) in a 2:1 volume ratio was used for preparing solution. In a typical procedure, Li_2_SO_4_ (65.96 mg), KH_2_PO_4_ (40.83 mg), MnSO_4_·H_2_O (50.71 mg), and KOH (0.2 mg) were sequentially dissolved in 30 mL of the EG/DI mixture under continuous magnetic stirring. The suspension was further stirred continuously for 60 minutes before being transferred into a 50-mL Teflon-lined stainless-steel autoclave for hydrothermal synthesis. The hydrothermal process was carried out in an oven at 180 °C for 12 hours. After cooling to room temperature, the synthesized products were centrifuged and washed several times with distilled water and ethanol.

*Figure S19: (a) cyclic-voltammetry profile at 1 mV s^-1^, and (b) galvanostatic charge-discharge profile at various current rates for LiMnPO_4_ cathode in 0.5 M AlCl_3_ electrolyte.*


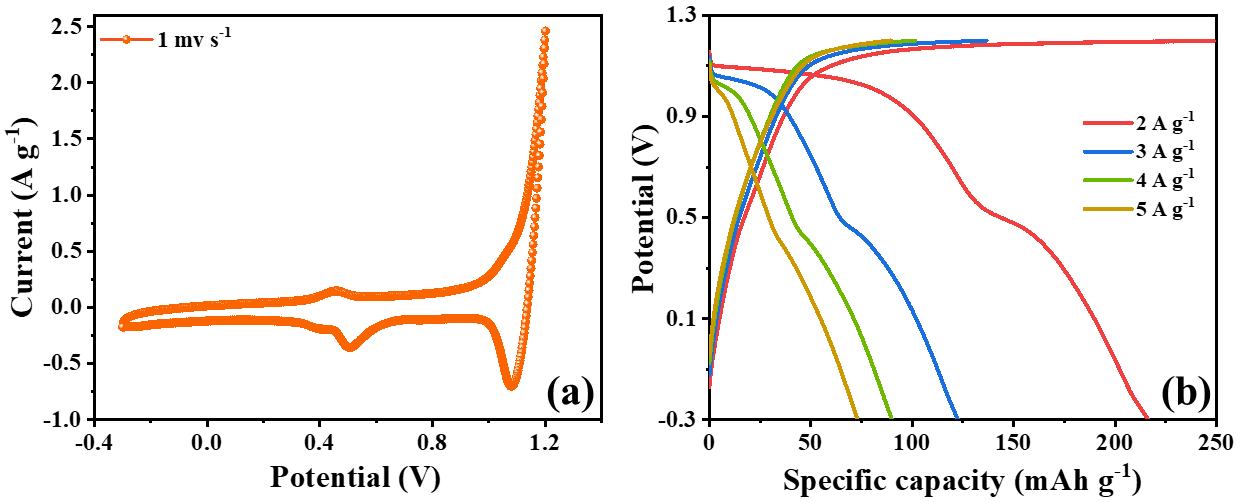


*The N/P ratio and the details of the charge balance calculation prior to device assembly:*

Charge balance calculation:

Prior to device assembly, charge balance was performed utilising the following mass balance relation:

$\frac{m_{+}}{m_{-}}=\frac{V_{-}C_{-}}{V_{+}C_{+}}$

where C_-_ and C_+_ are the capacity (in mAh g^-1^) measured using a three-electrode system at the same current density, and V_+_ and V_-_ signify the working potential windows for the cathode (positive electrode) and anode (negative electrode), respectively.

Positive electrode (Cathode):

$C_{+}=217 mAh g^{-1}$ at 2 A g^-1^ current density

$V_{+}=1.5 V$

Negative electrode (Anode):

$C_{-}=181 mAh g^{-1}$ at 2 A g^-1^ current density

$V_{-}=1.3 V$

Hence,

$\frac{m_{+}}{m_{-}}=\frac{V_{-}C_{-}}{V_{+}C_{+}}=\frac{1.3\times181}{1.5\times217}\cong0.7$

The N/P ration was calculated using the following equation:

$\frac{N}{P}=\frac{m_{-}C_{-}}{m_{+}C_{+}}$

where C_-_ and C_+_ are the capacity (in mAh g^-1^) measured using a three-electrode system at the same current density, and m_+_ and m_-_ denote the mass of the cathode (positive electrode) and anode (negative electrode), respectively.

Positive electrode (Cathode):

$C_{+}=217 mAh g^{-1}$ at 2 A g^-1^ current density

$m_{+}=1.05 mg$

Negative electrode (Anode):

$C_{-}=181 mAh g^{-1}$ at 2 A g^-1^ current density

$m_{-}=1.5 mg$

Hence,

$\frac{N}{P}=\frac{m_{-}C_{-}}{m_{+}C_{+}}=\frac{1.5\times181}{1.05\times217}\cong1.1$

References:

1. Yang, X.; Zhu, Z.; Dai, T.; Lu, Y., Facile Fabrication of Functional Polypyrrole Nanotubes via a Reactive Self‐Degraded Template. *Macromol. Rapid Commun.* **2005,** *26* (21), 1736-1740.

2. Blochl, P. E., Projector augmented-wave method. *Phys. Rev. B Condens. Matter.* **1994,** *50* (24), 17953-17979.

3. Kresse, G.; Joubert, D., From ultrasoft pseudopotentials to the projector augmented-wave method. *Phys. Rev. B* **1999,** *59* (3), 1758-1775.

4. Klimeš, J.; Bowler, D. R.; Michaelides, A., Van der Waals density functionals applied to solids. *Phys. Rev. B* **2011,** *83* (19), 195131.

5. Augustyn, V.; Simon, P.; Dunn, B., Pseudocapacitive oxide materials for high-rate electrochemical energy storage. *Energy Environ. Sci.* **2014,** *7* (5), 1597.

6. Nandi, S.; Das, S. K., An electrochemical study on bismuth oxide (Bi_2_O_3_) as an electrode material for rechargeable aqueous aluminum-ion battery. *Solid State Ion.* **2020,** *347*, 115228.

7. Elgrishi, N.; Rountree, K. J.; McCarthy, B. D.; Rountree, E. S.; Eisenhart, T. T.; Dempsey, J. L., A Practical Beginner’s Guide to Cyclic Voltammetry. *J. Chem. Educ.* **2017,** *95* (2), 197-206.

8. Luo, H.; Yu, P.; Li, G.; Yan, K., Topological quantum materials for energy conversion and storage. *Nat. Rev. Phys.* **2022,** *4* (9), 611-624.

9. Obeid, M. M.; Sun, Q., Recent advances in topological quantum anode materials for metal-ion batteries. *J. Power Sources* **2022,** *540*, 231655.

10. Wu, W.; Wang, S.; Sun, Q., Topological Quantum Cathode Materials for Fast Charging Li‐Ion Battery Identified by Machine Learning and First Principles Calculation. *Adv. Theory Simul.* **2022,** *5* (3), 2100350.

11. Han, D.; Cui, C.; Zhang, K.; Wang, Z.; Gao, J.; Guo, Y.; Zhang, Z.; Wu, S.; Yin, L.; Weng, Z.; Kang, F.; Yang, Q.-H., A non-flammable hydrous organic electrolyte for sustainable zinc batteries. *Nat. Sustain.* **2021,** *5* (3), 205-213.

12. Fei, H.; Yang, F.; Jusys, Z.; Passerini, S.; Varzi, A., Ethylene Glycol Co‐Solvent Enables Stable Aqueous Ammonium‐Ion Batteries with Diluted Electrolyte. *Adv. Funct. Mater.* **2024,** *34* (42), 2404560.

13. Zhu, Y.; Lao, Z.; Zhang, M.; Hou, T.; Xiao, X.; Piao, Z.; Lu, G.; Han, Z.; Gao, R.; Nie, L.; Wu, X.; Song, Y.; Ji, C.; Wang, J.; Zhou, G., A locally solvent-tethered polymer electrolyte for long-life lithium metal batteries. *Nat. Commun.* **2024,** *15* (1), 3914.

14. Ren, H.; Li, S.; Xu, L.; Wang, L.; Liu, X.; Wang, L.; Liu, Y.; Zhang, L.; Zhang, H.; Gong, Y.; Lv, C.; Chen, D.; Wang, J.; Lv, Q.; Li, Y.; Liu, H.; Wang, D.; Cheng, T.; Wang, B.; Chao, D.; Dou, S., Tailoring Water-in-DMSO Electrolyte for Ultra-stable Rechargeable Zinc Batteries. *Angew. Chem. Int. Ed. Engl.* **2025,** *64* (13), e202423302.

15. Salomon, M., Solubility problems relating to lithium battery electrolytes. *Pure Appl. Chem.* **1998,** *70* (10), 1905-1912.

16. Zhao, H.; Liu, H.; Qu, J., Effect of pH on the aluminum salts hydrolysis during coagulation process: formation and decomposition of polymeric aluminum species. *J. Colloid. Interface. Sci.* **2009,** *330* (1), 105-12.

17. De, P.; Halder, J.; Priya, S.; Srivastava, A. K.; Chandra, A., Two-Dimensional V_2_O_5_ Nanosheets as an Advanced Cathode Material for Realizing Low-Cost Aqueous Aluminum-Ion Batteries. *ACS Appl. Energy Mater.* **2023,** *6* (2), 753-762.

18. De, P.; Bharti, L.; Halder, J.; Priya, S.; Chandra, A., Electrochemically activated Mn_3_O_4_ nanoparticles as higher performing electrode than MnO_2_ for Al-ion batteries – An insight into the crystallographic changes caused by Al^3+^ intercalation. *Electrochim. Acta* **2023,** *469*, 143248.

19. Zhao, A.; Peng, J.; Mao, W.; Wang, Q.; Zhu, Y.; Peng, N., Fe-Co PBA/rGO interface strategy enabling fast Al^3+^ intercalation for stable aqueous Al-ion batteries. *J. Chem. Eng.* **2024,** *493*, 152790.

20. De, P.; Priya, S.; Halder, J.; Srivastava, A. K.; Chandra, A., Metal-Organic Framework for Aluminum based Energy Storage Devices: Utilizing Redox Additives for Significant Performance Enhancement. *ACS Appl. Mater. Interfaces* **2024,** *16* (20), 26299-26315.

21. Baishya, R.; Phukon, H.; Kalita, D.; Barman, S. R.; Das, S. K., Investigation on Al^3+^ ion storage in Bi_2_MoO_6_ and Bi_2_WO_6_ for rechargeable aqueous aluminum-ion battery. *J. Energy Storage* **2024,** *94*, 112541.

22. Huang, C.; Jiang, Z.; Liu, F.; Li, W.; Liang, Q.; Zhao, Z.; Ge, X.; Song, K.; Zheng, L.; Zhou, X.; Qiao, S.; Zhang, W.; Zheng, W., Oxygen Vacancies Boosted Hydronium Intercalation: A Paradigm Shift in Aluminum-Based Batteries. *Angew. Chem. Int. Ed. Engl.* **2024,** *63* (26), e202405592.

23. Nandi, S.; Goswami, T. K.; Das, S. K., Al^3+^ ion storage in LiMnPO_4_ for rechargeable aqueous aluminum-ion battery. *Mater. Lett.* **2023,** *346*, 134513.

24. Unal, B.; Sel, O.; Demir-Cakan, R., Current collectors corrosion behaviours and rechargeability of TiO_2_ in Aqueous Electrolyte Aluminium-ion batteries. *J. Appl. Electrochem.* **2023,** *54* (6), 1425-1434.

25. Nandi, S.; Das, S. K., An electrochemical study on LiMn_2_O_4_ for Al^3+^ ion storage in aqueous electrolytes. *Phys. Chem. Chem. Phys.* **2021,** *23* (35), 19150-19154.

26. Joseph, J.; Nerkar, J.; Tang, C.; Du, A.; O'Mullane, A. P.; Ostrikov, K. K., Reversible Intercalation of Multivalent Al^3+^ Ions into Potassium-Rich Cryptomelane Nanowires for Aqueous Rechargeable Al-Ion Batteries. *ChemSusChem* **2019,** *12* (16), 3753-3760.

27. Lahan, H.; Boruah, R.; Hazarika, A.; Das, S. K., Anatase TiO_2_ as an Anode Material for Rechargeable Aqueous Aluminum-Ion Batteries: Remarkable Graphene Induced Aluminum Ion Storage Phenomenon. *J. Phys. Chem. C* **2017,** *121* (47), 26241-26249.

28. Kumar, S.; Satish, R.; Verma, V.; Ren, H.; Kidkhunthod, P.; Manalastas, W.; Srinivasan, M., Investigating FeVO_4_ as a cathode material for aqueous aluminum-ion battery. *J. Power Sources* **2019,** *426*, 151-161.

29. Lahan, H.; Das, S. K., Reversible Al^3+^ ion insertion into tungsten trioxide (WO_3_) for aqueous aluminum-ion batteries. *Dalton Trans.* **2019,** *48* (19), 6337-6340.

30. Liu, S.; Pan, G. L.; Li, G. R.; Gao, X. P., Copper hexacyanoferrate nanoparticles as cathode material for aqueous Al-ion batteries. *J. Mater. Chem. A* **2015,** *3* (3), 959-962.

31. Bao, L.; Chen, Y.; Xu, G.; Yang, T.; Ji, Z., Hydrothermal Synthesis of Monodispersed LiMnPO_4_ (010) Nanobelts and [001] Nanorods and Their Applications in Lithium‐Ion Batteries. *Eur. J. Inorg. Chem.* **2018,** 1533-1539. (DOI: 10.1002/ejic.201701270)
